# A Genomic Instability-Related Long Noncoding RNA Signature for Predicting Hepatocellular Carcinoma Prognosis

**DOI:** 10.1155/2022/3090523

**Published:** 2022-08-29

**Authors:** Jing Lu, Wanyue Cao, Zeping He, Haoyu Wang, Jialing Hao, Junming Xu

**Affiliations:** Department of General Surgery, Shanghai General Hospital, School of Medicine, Shanghai Jiaotong University, Shanghai 200080, China

## Abstract

**Background:**

Long noncoding RNAs (lncRNAs) are found to be novel biomarkers for hepatocellular carcinoma (HCC) and play an important role in tumor progression. We established a genomic instability-related long noncoding RNA signature (GIlncSig) as an independent prognosis factor and also investigated its impact on prognosis significance.

**Method:**

Somatic mutation profiles, clinical characteristics, and RNA sequencing data were obtained from The Cancer Genome Atlas (TCGA) database. Lasso regression was used to construct GIlncSig. KEGG was used to identify the possible biological pathways. ESTIMATE and CIBERSORT algorithms were used to calculate the immune microenvironment scores and proportion of immune cells in HCC patients. The expression of LINC00501 was conducted by qRT-PCR. Cell proliferation was measured by EdU, CCK-8, and colony formation assay, and cell migration and invasion ability were measured by wound healing and transwell assay.

**Results:**

135 genomic instability-related lncRNAs were identified, and GIlncSig was constructed using 13 independent lncRNAs with significant prognosis values. Based on the GIlncSig, high-risk group had worse clinical outcomes than low-risk group, while high-risk group also had higher UBQLN4, KRAS, ARID1A, and PIK3CA expression. Moreover, the efficiency of GIlncSig combining single-gene mutation was higher than single-gene mutation alone such as TP53. The results of CIBERSORT and ESTIMATE showed that GS group and GU group had significantly different immune infiltration. In addition, LINC00501 was identified as a potential biomarker in HCC with strong relationship with clinical characteristics. In vitro assays validated that LINC00501 promoted proliferation and migration of HCC cell lines.

**Conclusion:**

Our results showed that GIlncSig serves as a potential independent prognosis factor to predict HCC patients' prognosis for exploring potential mechanism and therapy strategy. Besides, LINC00501 plays an important role in the progression of HCC, which may be a potential therapy target.

## 1. Introduction

Liver cancer is the sixth most common cancer worldwide and the fourth cause of cancer-related death. Hepatocellular carcinoma accounts for 90% of liver cancer. Hepatitis B virus, hepatitis C virus, and alcoholic and nonalcoholic fatty liver diseases are the most common risk factors of HCC [1]. Liver transplantation and liver resection are the most effective treatments for HCC therapy [2]; however, many patients have already lost surgical indications when they are diagnosed as HCC because the symptoms of HCC are always appearing late. Imaging techniques such as CT and MRI are the most effective ways to diagnose HCC [3]. Alpha fetoprotein (AFP), as a serum biomarker, is activated in up to 70% of HCC patients and associated with poor survival rates [4, 5]. However, a few HCC patients do not have elevated serum AFP level, so it cannot be used as a biomarker to predict recurrence [6]. The current biomarkers are limited by their specificity and sensitivity, and novel biomarkers are urgently to be discovered.

Long noncoding RNA (lncRNA) is a kind of RNA longer than 200 nucleotides, which is not translated into proteins [7]. LncRNAs play an important role in gene regulation by interacting with DNA, RNA, or proteins [8]. LncRNAs have been found differently expressed in various cancers such as gastric carcinoma [9], colorectal carcinoma [10], and breast cancer [11], and lncRNAs are associated with different outcomes. Studies showed that lncRNAs may serve as new biomarkers to predict outcomes of HCC patients. Lnc-APUE is upregulated in HCC patients and promotes G1/S phase transition and tumor growth. Lnc-APUE is also associated with short recurrence-free survival [12]. Lnc-GAN1 is markedly downregulated in non-small-cell lung cancer and acts as a tumor suppressor [13].

Genomic instability in different types of cancers is associated with a greater tendency to accumulate DNA damage [14]. Genomic instability may serve as a major driving force of tumorigenesis and associated with poor prognosis of cancer patients [15]. In addition, it can be a prognosis marker to predict outcomes.

In this study, lncRNAs and genomic instability were combined to establish the genomic instability-related long noncoding RNAs signature based on TCGA database to predict the prognosis of HCC patients. In addition, we also analyzed the KEGG pathway and immune infiltration in order to explore the mechanism of genomic instability lncRNAs. Furthermore, we validated the function of LINC00501 with CCK-8, EdU, colony formation, wound healing, and transwell assay.

## 2. Materials and Methods

### 2.1. Data Collection

The RNA sequencing data and somatic mutation profiles were obtained from TCGA database. 364 patients with full RNA-seq and somatic mutation profiles were used for further analysis. Pathological and clinical characteristics were also obtained from TCGA. After integrating 364 samples, 11 samples were excluded because of lacking of corresponding clinical characteristics. The concrete information of 353 samples is shown in Table S1.

### 2.2. Identification and Establishment of GI-Related lncRNAs

According to somatic mutation profiles, the cumulative somatic mutation was calculated. The top 25% of somatic mutation was defined as high-mutation group (HM group), while the bottom 25% of somatic mutation was defined as low-mutation group (LM group). The expression of different lncRNAs was conducted by Wilcoxon test, while |logFC| > 0.9 and *P*-value <0.05 were defined as the criteria of differently expressed lncRNAs. Unsupervised hierarchical clustering was used for all samples, and 353 samples were divided into two groups.

### 2.3. KEGG Enrichment Analysis

Pearson correlation coefficients were calculated between lncRNAs and mRNAs, and the top 10 protein-coding genes, which have strongest correlation with lncRNAs, were screened. KEGG enrichment analysis was applied to identify the possible biological pathways associated with lncRNAs in order to predict the function of lncRNAs.

### 2.4. Immune Infiltration Analysis

CIBERSORT is used for characterizing cell composition of complex tissues from gene expression profiles [16]. The quantity of 22 immunocyte subtypes can be obtained using CIBERSORT algorithm. We uploaded the expression of 353 HCC samples, setting the algorithm to 500 rows. In addition, the expression of immune checkpoint genes between GU group and GS group was compared.

### 2.5. Stromal Cell Analysis

Stromal cells have important role in tumor growth and disease progression. Immune score and stromal scores were calculated by ESTIMATE algorithm with the “estimate” package by R software. To explore the relationship between genomic instability and stromal cells infiltration, Wilcoxon *t*-test was conducted between GU group and GS group.

### 2.6. HCC Clinical Specimen Collection

We collected 33 pairs of HCC tissues and paired adjacent normal tissues from patients in Shanghai General Hospital between January 2015 and December 2020. All patients did not receive chemotherapy, radiotherapy, immunotherapy, or other therapies before surgery. This research was approved by the Ethics Committee of Shanghai General Hospital, and informed consents were obtained from all HCC patients.

### 2.7. Cell Culture and Transfection

The hepatocellular carcinoma cell line Huh-7 was obtained from Shanghai Cell Bank of Chinese Academy of Sciences (Shanghai, China). Dulbecco's modified Eagle's medium (Gibco, USA) containing 10% fetal bovine serum and 1% penicillin streptomycin (Gibco, USA) was used to culture cell. Cells were grown under an atmosphere containing 5% CO2 at 37°C.

Oligonucleotides were constructed to regulate LINC00501. The siRNA targeting LINC00501 was designed by Gene Pharma (Shanghai, China) as follows: 5′-CUGCGGAUGAACUGAAUAATT-3′(sense) and 5′-UUAUUCAGUUCAUCCGCAGTT-3′(antisense). The oligonucleotide was transfected into cells with Lipofectamine 2000 (Invitrogen, USA).

### 2.8. Quantitative Real-Time PCR (qRT-PCR)

Total RNAs from the tissue samples and HCC cell lines were extracted using Trizol (Takara Biotechnology, Japan). We used a reverse transcription kit (EnzyArtisan, China) to synthesize cDNA for subsequent PCR assay. QRT-PCR was performed with 2 × S6 Universal SYBR qPCR Mix (EnzyArtisan, China). The relative mRNA expression levels were normalized to GAPDH and calculated by the 2^−ΔΔct^ method. The forward primer of LINC00501 is CCCTGTTCTCCCAAGTGCAA, and the reverse primer is CCTACTGTGGCTAACGAGCA.

### 2.9. CCK-8 Assay, EdU Assay, and Colony Formation Assay

Cells were cultured in 96-well plates at 5000 cells per well. Cell proliferation was measured by the Cell Counting Kit-8 (CCK-8) assay (NCM Biotech, China). The absorbance at 450 nm was observed at 24 h and 48 h.

Cells were cultured in 96-well plates and then incubated with the Cell-Light EdU Apollo 567 (RiboBio, China) for 2 hours.

Cells were cultured in 6-well plates at 5000 cells per well. Cells were washed with PBS for three times and fixed with 4% paraformaldehyde after 2 weeks. Then, cells were stained with crystal violet solution and photographed.

### 2.10. Wound Healing Assay and Transwell Assay

Cells were cultured to 90% confluence in 6-well plates. Sterile 200-*μ*L pipette tips were used to scratch the cell layers. After washing three times with PBS, serum-free DMEM was added. An inverted microscope was used to observe and photograph the cells at 0 h, 24 h, and 48 h, respectively.

600-*μ*L DMEM containing 10% FBS was added to the lower chamber, while 200-*μ*L serum-free DMEM with cells was added into the upper transwell chamber. After 24 h of culturing, cells were fixed by paraformaldehyde. Cells on the underside of the transwell chamber membrane were stained with 0.1% crystal violet and photographed.

### 2.11. Statistical Analysis

Univariate Cox regression was conducted to determine lncRNAs associated with overall survival rates. Furthermore, Lasso regression was conducted to establish a GIlncSig in order to predict the outcomes of HCC patients. GIlncSig can be described as follows:

(1) $$\begin{array}{c} \text{GIlncSig}\left(\text{patients}\right)=\sum_{i=1}^{n}\text{exp}\left(\text{lncRNAi}\right)*\text{coef}\left(\text{lncRNAi}\right). \end{array}$$

GIlncSig (patients) represents the predicted risk score of each patient, and exp (lncRNAi) is the expression level of lncRNAs in each patient. Coef (lncRNAi) is the contribution index of each lncRNA to predicted risk score.

353 samples were divided into high-risk group and low-risk group according to the cutoff of median risk score. Kaplan–Meier method was used to calculate the survival rate of two groups. Univariate and multivariate Cox regression was used on age, gender, stage, grade, T stage, N stage, M stage, and risk score to assess the prognosis value of GIlncSig. R-version (v.4.0.2) software was used to perform statistical analysis with statistical methods. All experiments were replicated three times.

## 3. Results

### 3.1. Identification of GI-Related LncRNAs in Hepatocellular Carcinoma

**Figure 1:**
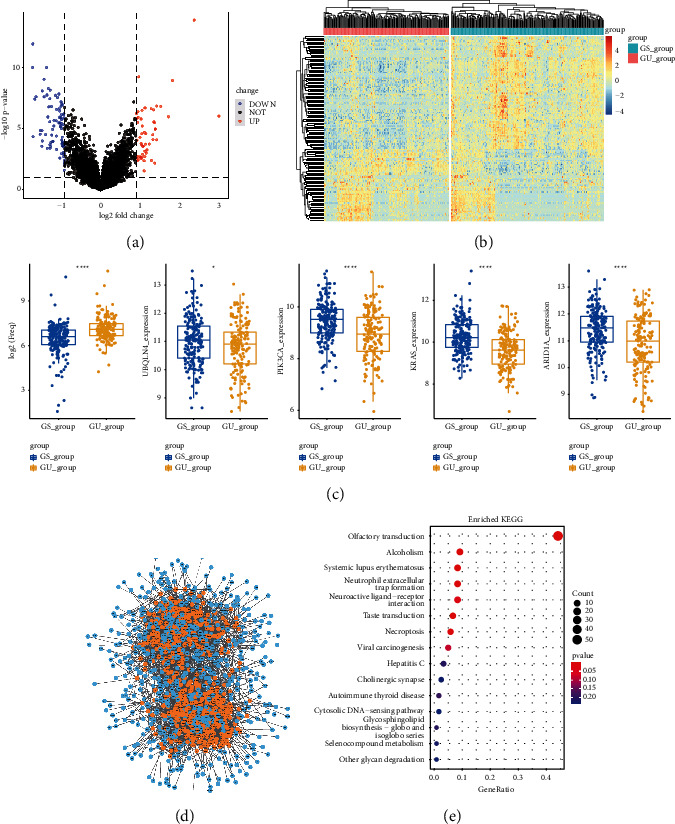
Identification of GI-related lncRNAs in HCC. (a) Volcano plot of differently expressed lncRNAs. The right orange labeled lncRNAs were significantly higher expressed in HM group, while the left blue labeled lncRNAs were significantly low expressed in HM group. (b) Heatmap of 353 HCC samples according to the expression of 135 GI-related lncRNAs. The right cluster was GS group and the left cluster was GU group. (c) Somatic mutation counts were significantly higher expressed in GU group, while the expression of UBQLN4, KRAS, PIK3CA, and ARID1A was significantly higher expressed in GS group. (d) Co-expression network of mRNAs and lncRNAs. The orange circles represent lncRNAs, and blue circles represent mRNAs. (e) KEGG analysis of lncRNAs co-expressed mRNAs.

According to the cumulative number results based on somatic mutations of 353 samples from TCGA, HM group was defined as the top 25 percent (*n* = 88) of highest somatic mutations and LM group was defined as the last 25 percent (*n* = 88) of lowest somatic mutations (Table S2). Different expression gene analysis showed that 135 lncRNAs were significantly changed between HM group and LM group with |log2FC| > 0.9 and *P*-value <0.05. The volcano plot showed that 52 lncRNAs were upregulated and 83 lncRNAs were downregulated (Figure 1(a), Table S3). Based on the 135 genomic instability lncRNAs, unsupervised hierarchical clustering divided 353 samples into two groups named genomic unstable group (GU group, *n* = 159) and genomic stable group (GS group, *n* = 194) (Figure 1(b), Table S4). GU group had higher cumulative somatic mutation counts than GS group. The expression of UBQLN4, KRAS, ARID1A, and PIK3CA was significantly differently expressed in GU group and GS group (Figure 1(c), *P* < 0.05). Pearson correlation coefficients were calculated between lncRNAs and mRNAs in order to validate the potential function of these lncRNAs, and the top 10 protein-coding genes, which have strongest correlation with lncRNAs, were screened. An lncRNA-mRNAco-expression network was constructed using this connection (Figure 1(d)). In order to explore the potential function of these mRNAs, functional analysis was performed. KEGG analysis revealed that the important pathways include alcoholism, neuroactive ligand-receptor interaction, cytokine-cytokine receptor interaction, and autoimmune thyroid disease (Figure 1(e)). These pathways indicated that these lncRNAs have a strong correlation with immunity.

### 3.2. Establishment of the GI-Related LncRNAs Signature

**Figure 2:**
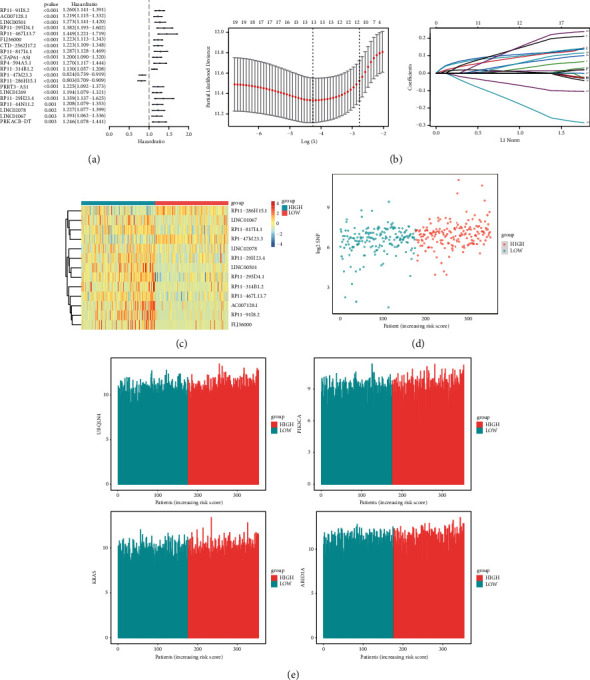
Establishment of the GI-related lncRNAs signature. (a) Univariate cox regression analysis of 20 genomic instability-related lncRNAs. (b) Lasso regression model for 20 lncRNAs. (c) heatmap of the GIlncSig. The right cluster was low-risk group, and the left cluster was high-risk group. (d) Distribution of somatic mutation. (e) Distribution of UBQLN4, KRAS, PIK3CA, and ARID1A.

Univariate cox regression was performed in order to determine the prognosis value of these lncRNAs. Among the identified 135 lncRNAs, 20 lncRNAs have the strongest correlation with overall survival of 353 samples (Figure 2(a)). Then, Lasso regression analysis was performed. A GIlncSig was constructed according to the coefficients and the expression of 13 lncRNAs (Figure 2(b)). The specific formula of the GIlncSig was as follows:

(2) $$\begin{array}{c} \text{GIlncSig}=\left(0.0029*\text{RP}11-91\text{I}8.2\right)+\left(0.1078*\text{AC}007128\text{.}1\right)+\left(0.0421*\text{LINC}00501\right)+\left(0.1156*\text{RP}11-295\text{D}4\text{.}1\right)+\left(0.1132*\text{RP}11-467\text{L}13\text{.}7\right)+\left(0.0131*\text{FLJ}36000\right)+\left(0.1791*\text{RP}11-817\text{I}4\text{.}1\right)+\left(0.0719*\text{RP}11-314\text{B}1\text{.}2\right)+\left(-0.2280*\text{RP}1-47\text{M}23\text{.}3\right)+\left(-0.0893*\text{RP}11-286\text{H}15\text{.}1\right)+\left(0.0014*\text{RP}11-29\text{H}23\text{.}4\right)+\left(0.0837*\text{LINC}02078\right)+\left(0.1974*\text{LINC}01067\right). \end{array}$$

According to the GIlncSig scores calculated from the model, 353 samples were divided into high-risk group and low-risk group via the middle risk score (Table S5). As shown in Figure 2(c), lncRNA RP11-91I8.2, AC007128.1, LINC00501, RP11-295D4.1, RP11-467L13.7, FLJ36000, RP11-817I4.1, RP11-314B1.2, RP11-29H23.4, LINC02078, and LINC01067 showed upregulated expression in high-risk group, while lncRNA RP1-47M23.3 and RP11-286H15.1 showed downregulated expression in high-risk group. Somatic mutation counts and the expression of UBQLN4, KRAS, ARID1A, and PIK3CA along with increasing GIlncSig scores are displayed (Figures 2(d) and 2(e)).

### 3.3. Evaluation of the GI-Related LncRNAs Signature

**Figure 3:**
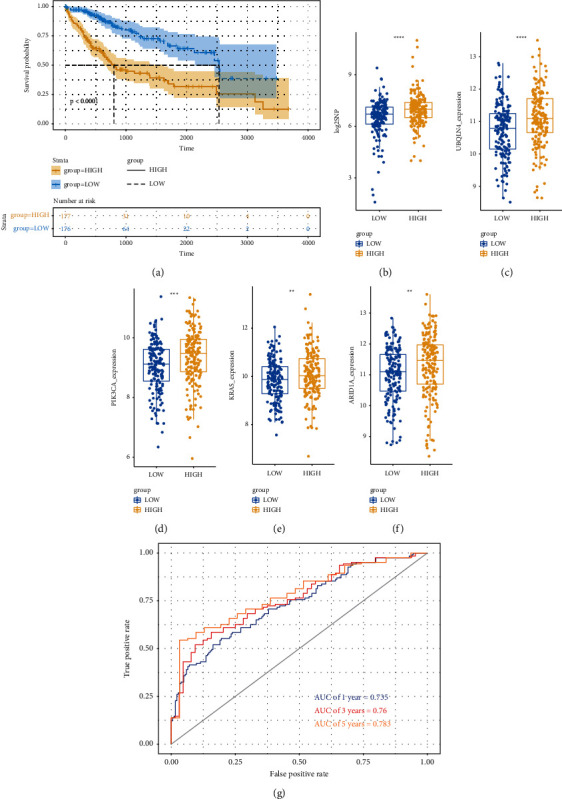
Evaluation of the GIlncSig efficacy. (a) K-M analysis showed that low-risk group had higher overall survival than high-risk group. (b) High-risk group has higher cumulative somatic mutation counts than low-risk group. ((c)-(f)) The expression of UBQLN4, KRAS, ARID1A, and PIK3CA was higher in high-risk group. (g) 1-, 3-, and 5-year ROC curve analysis of the GIlncSig.

The Kaplan–Meier analysis showed that high-risk group had lower overall survival than low-risk group (Figure 3(a)). High-risk group has higher cumulative somatic mutation counts than low-risk group (Figure 3(b)). In high-risk group, the expression of UBQLN4, which is a genomic instable driver gene, was also upregulated compared with low-risk group (Figure 3(c)). In addition, the expression of KRAS, ARID1A, and PIK3CA was also differently expressed between high-risk group and low-risk group (Figures 3(d)–3(f)). ROC curve was used to assess the credibility of the model, and the area under the curve was 0.735 (1 year), 0.76 (3 years), and 0.783 (5 years) (Figure 3(g)).

### 3.4. Independent Validation of GI-Related LncRNAs Signature from Clinical Factors

**Figure 4:**
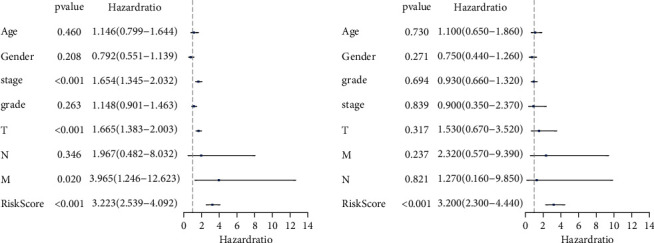
Univariate and multivariate cox regression of clinical factors and risk score.

**Figure 5:**
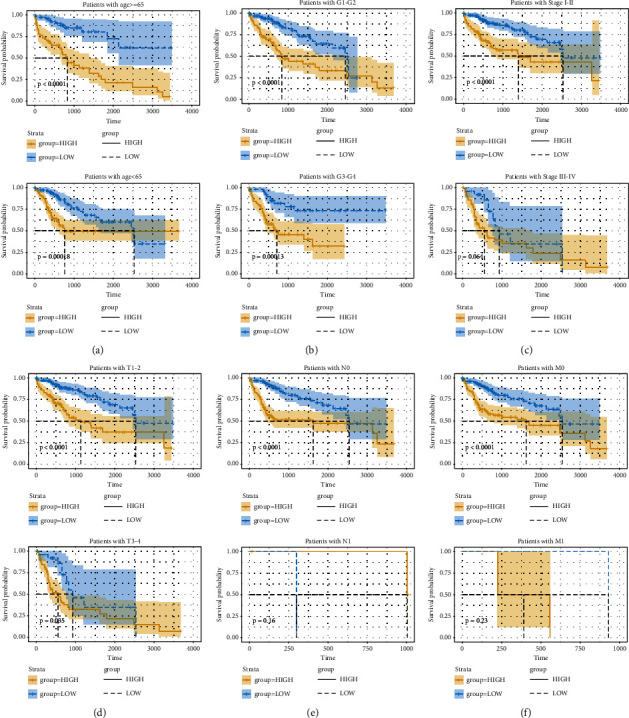
Evaluation of GIlncSig efficacy in clinical factors. (a) K-M analysis of os of patients with high- or low-risk scores with age < 65 or age ≥ 65. (b) K-M analysis of overall survival of patients with high- or low-risk scores with G1-2 or G3-4. (c) K-M analysis of os of patients with high- or low-risk scores with stage I-II or stage III-IV. (d) K-M analysis of os of patients with high- or low-risk scores with T1-2 or T3-4. (e) K-M analysis of os of patients with high- or low-risk scores with N0 or N1. (f) K-M analysis of os of patients with high- or low-risk scores with M0 or M1.

**Figure 6:**
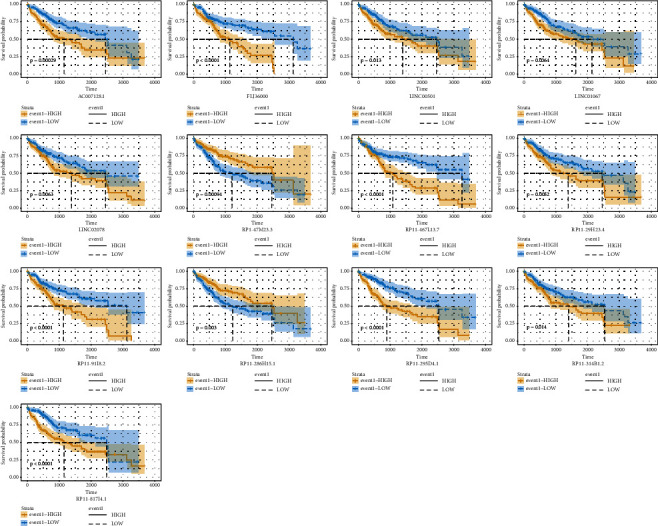
Overall survival of lncRNAs in GIlncSig. K-M analysis showed that 13 lncRNAs were correlated with os of HCC patients.

**Figure 7:**
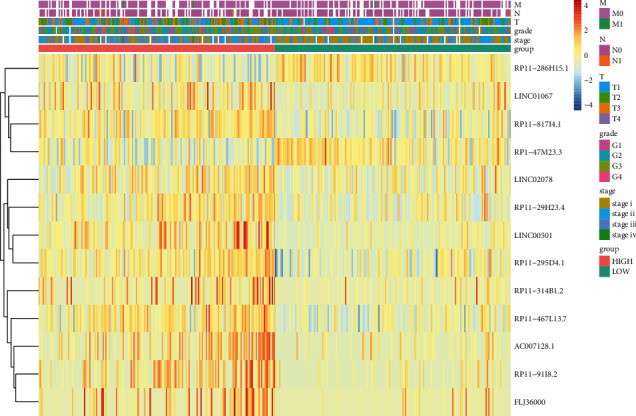
Clinical characteristics of lncRNAs in GIlncSig.

In order to assess the prognosis value of clinical factors, univariate and multivariate Cox regression analyses were used on gender, age, stage, grade, T stage, N stage, M stage, and risk score. Among these results, stage (HR = 1.654, 95% CI: 1.345–2.032, *P* < 0.001), T (HR = 1.665, 95% CI: 1.383–2.003, *P* < 0.001), M (HR = 3.965, 95% CI: 1.246–12.623, *P* < 0.020), and risk score (HR = 3.223, 95% CI: 2.539–4.092, *P* < 0.001) showed significant independent prognosis values in univariate cox regression, while only risk score (HR = 3.200, 95% CI: 2.300–4.440, *P* < 0.001) showed significant independent prognosis values in multivariate Cox regression (Figure 4, Table S6). In order to examine whether the prognosis performance of the GIlncSig was independent of any other clinical factors, the clinical factors were divided into different groups including high-risk group and low-risk group, female and male, age < 65 and age ≥ 65, G1-2 and G3-4, stage I-II and stage III-IV, T1-2 and T3-4, N0 and N1, and M0 and M1. A significant difference between these groups was observed among age, grade, stage, T, N, and M (Figures 5(a)–5(f)), which indicated that GIlncSig could be an independent prognosis factor for os of HCC. K-M analysis showed that 11 lncRNAs were risk factors and 2 lncRNAs were protective factors. K-M analysis results were corresponded to coefficients in GIlncSig (Figure 6). The correlation between 13 lncRNAs and clinical factors in TCGA datasets was represented by heatmap (Figure 7).

### 3.5. Comparison of GI-Related LncRNAs Signature with Single-Gene Mutation in Prognosis Value

**Figure 8:**
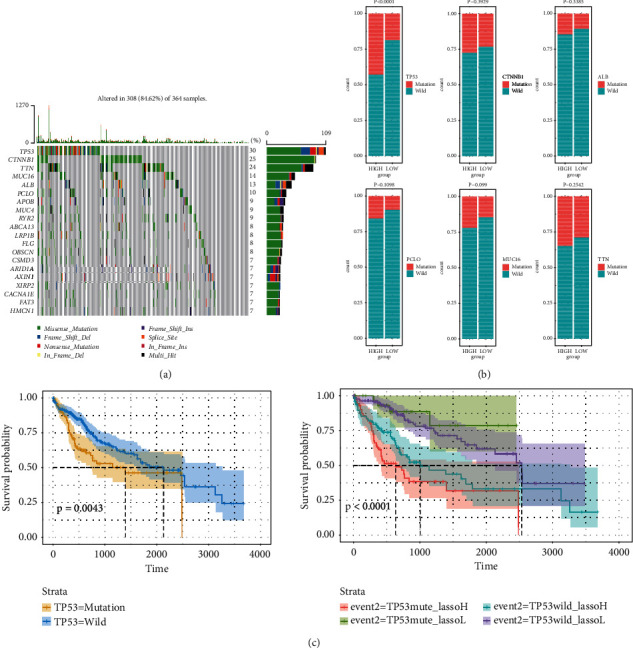
Comparison of GIlncSig with single-gene mutation. (a) Waterfall plot of the 20 most frequently mutated genes. (b) The proportion of top 6 genes mutation between high-risk and low-risk group. (c) K-M analysis of overall survival with different combinations of TP53 and GIlncSig.

In order to assess the prognosis value of GIlncSig and single-gene mutation, top six genes, which were frequently mutated, were selected including TP53, CTNNB1, TTN, MUC16, ALB, and PCLO (Figure 8(a)). High-risk group had significantly higher TP53 mutation rate (42.94%) compared with low-risk group (18.97%), which suggested that GIlncSig could be a predictive factor for mutation (Figure 8(b)). In order to assess the efficiency of GIlncSig, we combined GIlncSig and TP53 mutation, classifying patients into TP53 mutation/high-risk group, TP53 wild/high-risk group, TP53 mutation/low-risk group, and TP53 wild/low-risk group. K-M analysis indicated that the curves of these four groups were remarkable different. Patients who combined with TP53 mutation/low-risk group had remarkable higher os rate compared with patients combined with TP53 mutation/high-risk group, and patients who combined with TP53 wild/low-risk group had remarkable higher os rate compared with patients combined with TP53 wild/high-risk group (Figure 8(c)). In consequence, GIlncSig combined with gene mutation information had better prognosis value than single-gene mutation alone.

### 3.6. Genomic Instability Had a Strong Relationship with Immune Infiltration in Hepatocellular Carcinoma Samples

**Figure 9:**
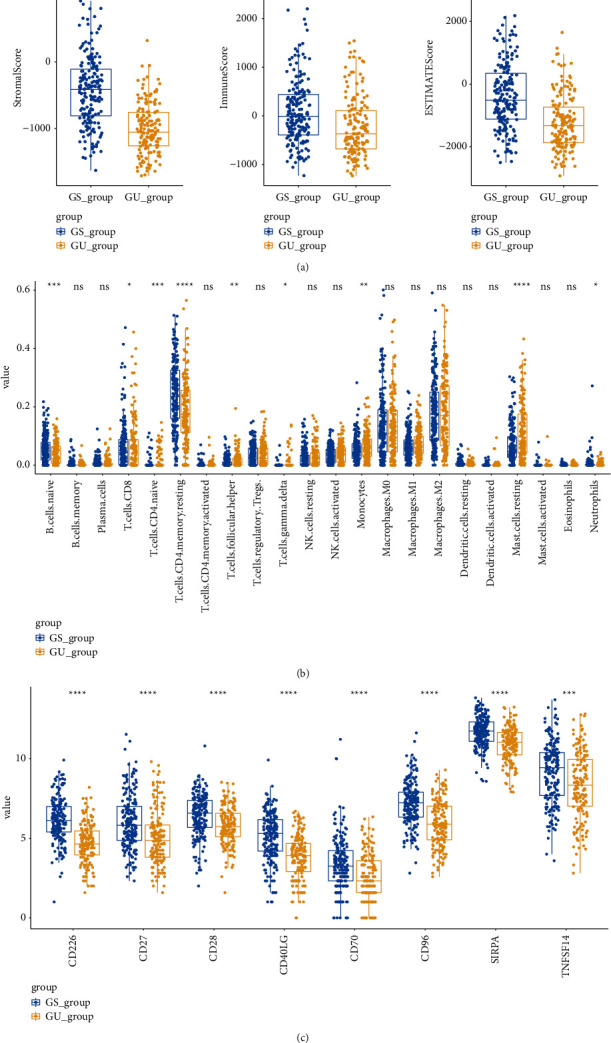
Identification of immune microenvironment and genomic instability of HCC. (a) The expression of immune scores, ESTIMATE scores, and stromal scores in GS group was higher than GU group. (b) The proportion of 22 types of immune cells infiltrating in HCC samples between GU group and GS group. (c) The expression of immune checkpoints between GS group and GU group.

In order to assess the relationship between genomic instability and immune infiltration in HCC, ESTIMATE was performed to compute immune scores, ESTIMATE scores, and stromal scores. GU group had significantly lower scores compared with GS group in these three indices (Figure 9(a)). Furthermore, CIBERSORT was performed to calculate the proportion of 22 types of immune cells infiltrating in HCC tissues. The results showed that GU group had significantly higher proportion of follicular helper T cells, monocytes, CD4 naïve T cells, gamma delta T cells, and resting mast cells than GS group and GS group had significantly higher proportion of naïve B cells, resting memory CD4+T cells, CD8+T cells, and neutrophils (Figure 9(b)). Also, the expression of immune checkpoint genes between GS group and GU group was analyzed. The results showed there were significant differences among CD226, CD27, CD28, CD40LG, CD70, CD96, SIRPA, and TNFSF14 (Figure 9(c)).

### 3.7. Nomogram Was Performed to Predict the Value of the GI-Related LncRNAs Signature

**Figure 10:**
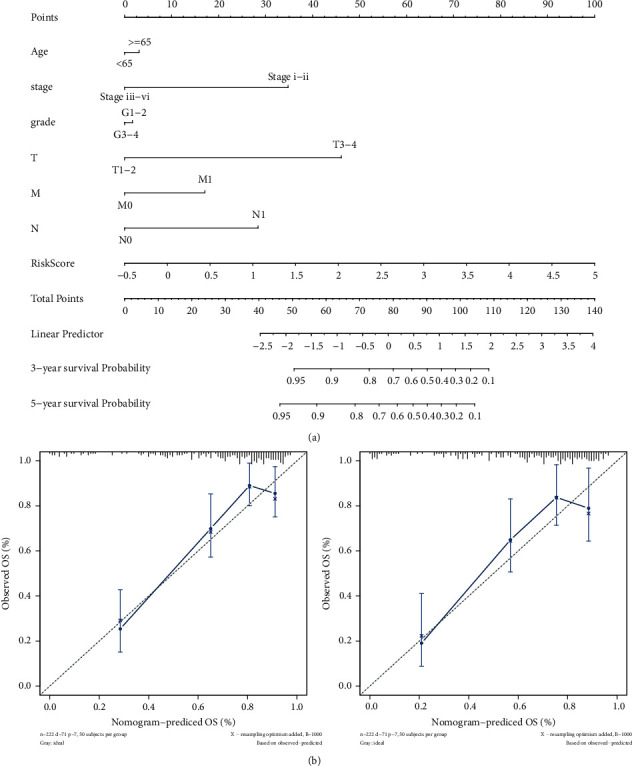
(a) Nomogram combing clinical factors and risk score. (b) Calibration curves illustrated the consistency between predicted and observed 3-year and 5-year survival rates depending on the prognostic nomogram.

A nomogram was constructed to reveal the 3- and 5-year survival rates in order to assess the prediction value of GIlncSig based on risk score and clinical factors including age, stage, grade, T stage, N stage, and M stage (Figure 10(a)). Calibration plots were used to compare the consistency of the actual and the predicted 3-year and 5-year patient survival (Figure 10(b)). The results suggested that nomogram was an efficient tool to predict prognosis.

### 3.8. LINC00501 Had a Strong Impact on Hepatocellular Carcinoma

**Figure 11:**
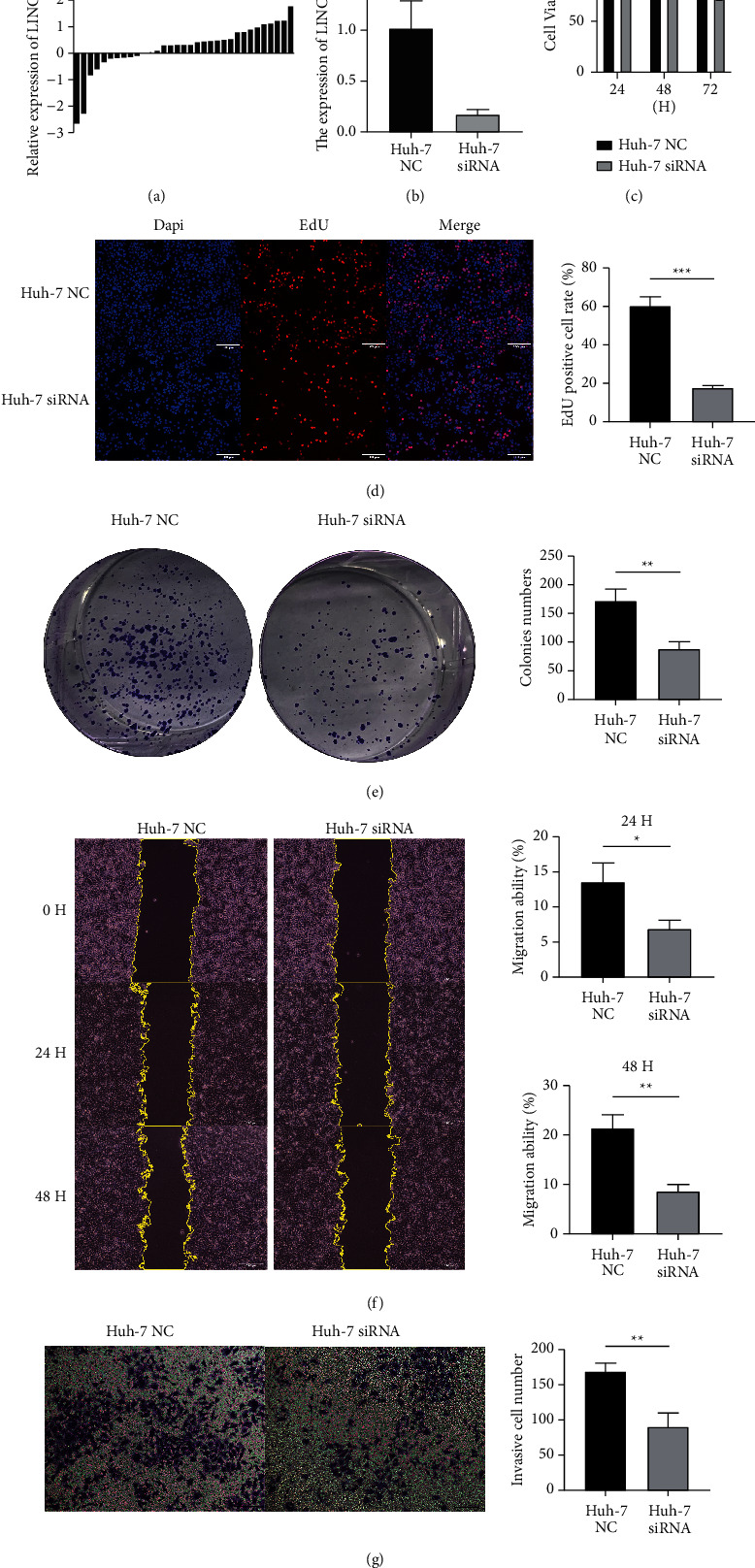
The expression and function of LINC00501 in HCC. (a) LINC00501 was highly expressed in HCC tissues compared with the adjacent normal tissues. (b) The efficiency of siRNA targeting LINC00501. ((c), (d), (e)) CCK-8, EdU, and colony formation assay indicated that LINC00501 knockdown inhibited proliferation of Huh-7. ((f), (g)) Wound healing and transwell assay indicated that LINC00501 knockdown inhibited migration of Huh-7. All experiments were replicated three times.

LINC00501 served as a risk factor according to GIlncSig and may play an important role in HCC progression. Therefore, we assessed the expression and function of LINC00501. We conducted qRT-PCR to analyze the expression of LINC00501 in 33 pairs of tissues. The results showed that LINC00501 was highly expressed in 23/33(69.7%) of the HCC tissues (Figure 11(a)). In order to assess the function of LINC00501, siRNA was transfected into Huh-7 to establish LINC00501 knockdown cell line (Figure 11(b)). CCK-8, EdU, and colony formation assay were used to assess the proliferation. CCK-8 assay (Figure 11(c)) showed that LINC00501 knockdown effectively inhibited the proliferation of Huh-7 transfected with siRNA. EdU assay and colony formation assay (Figures 11(d) and 11(e)) exhibited the same trend. Invasion and migration ability of LINC00501 were assessed by transwell and wound healing assay. The results indicated that the knockdown of LINC00501 decreased the migration and invasion ability, respectively (Figures 11(f) and 11(g)). Thus, LINC00501 knockdown can effectively inhibit proliferation and migration of Huh-7 cell line in vitro.

## 4. Discussion

During the diagnosis of HCC, imaging examination plays an important role; however, the lesion can be missed when it is too small. Pathological examination is still the standard of diagnosis, while the molecular biomarkers such as AFP are also widely used. Part of HCC patients has normal AFP values, while the specificity of AFP was 80–94% with a sensitivity of 41–65% [17]. It is urgent to find some new markers to predict survival rate of HCC patients.

Genomic instability has been recognized as the driver of carcinoma and plays a significant role in tumor progression [18]. Genomic instability has several complex mechanisms including DNA damage repair, DNA replication, and transcription [19]. A recent study showed that genomic instability can be a prognosis marker in pancreatic cancer [20].

Researches showed that lncRNAs play a significant role in different types of cancers. The mechanism of this function includes interacting with protein, RNA, and DNA [21]. Study showed that lncRNA is upregulated in colorectal cancer and correlated with poor prognosis [22]. Wu's research focused on the genomic instability-related lncRNAs in HCC and established a prognosis model consisting of 4 lncRNAs [23]. However, the relationship and mechanism between genomic instability and lncRNAs in tumor prognosis were largely ignored; therefore, we explored whether GI-related lncRNAs are associated with tumorigenesis and prognosis.

In this study, 135 novel genomic instability lncRNAs including 52 upregulated genes and 83 downregulated genes were screened. GS group and GU group were divided according to 135 lncRNAs. Somatic mutation and the expression of UBQLN4, KRAS, ARID1A, and PIK3CA were significantly differently expressed between these two groups. According to recent research, UBQLN4 deficiency leads to cellular sensitivity to genotoxic stress and is associated with genomic instability. Also, UBQLN4 is upregulated in various aggressive tumors and associated with poor outcomes [24, 25]. KRAS, ARID1A, and PIK3CA are three genes, which are frequently muted in human cancers worldwide [26]. The differences of these genes between these two groups suggested that the lncRNAs are correlated with genomic instability.

GIlncSig was established using lncRNAs with independent prognosis values according to univariate cox regression. According to GIlncSig predicted risk score, 353 patients were divided into two groups. Group with high-risk has significantly lower os rate than group with low risk. Clinical factors including age, stage, grade, T stage, N stage, and M stage were observed differently between these groups. Just as we suspected, high-risk group may indicate lower os, higher TNM stage, and advanced metastasis. TP53 mutation is the most common mutation in HCC, attributing to the poor prognosis of HCC and promoting the progression [27]. However, TP53 mutation alone may not able to predict the prognosis of HCC patients because of its instability and contingency, so GIlncSig combined with TP53 mutation information was constructed and showed better prognosis value than TP53 mutation alone.

In order to analyze whether genomic instability affects HCC through immunity, ESTIMATE algorithm and CIBERSORT were introduced. The results of ESTIMATE suggested that genomic instability may accelerate the progression of HCC by affecting tumor immune microenvironment. Furthermore, the relationship between genomic instability and the proportion of 22 types of immune cells was calculated. The results showed that several immune cells were differently infiltrated in HCC including CD4 naïve T cells, gamma delta T cells, follicular helper T cells, monocytes, CD8+T cells, resting mast cells, naïve B cells, resting memory CD4+T cells, and neutrophils. According to various researches, tumor-associated neutrophils have antitumor abilities including direct cytotoxicity and inhibition of tumors [28]. GS group also had a higher proportion of resting memory CD4+T cells and CD8+T cells, which may contribute to the antitumor ability. These also suggested that genomic instability represents poor immunity and promotes the progression of HCC through affecting tumor immune microenvironment. Tumor-associated immune cells play a significant role in tumor formation and progression and correlated with patient's overall survival. The relationship between genomic instability and immune cells could be a promising target for further investigation. The mechanism of how genomic instability affecting immune cells infiltrating tumors also needs to be studied.

Among the 13 lncRNAs, this study showed that AC007128.1 is upregulated in esophageal squamous cell carcinoma and associated with poor prognosis [29], while RP11-286H15.1 is proved significantly decreased in HCC and suggests a shorter survival time [30]. The results of these researches supported our GIlncSig and suggested that we could perform more research on these lncRNAs. Among 13 lncRNAs, LINC00501 was selected and molecular biology experiments in vitro were conducted. LINC00501 is a new lncRNA, and there is no study on HCC previously. Research showed that LINC00501 is highly expressed in non-small-cell lung cancers and patients with high LINC00501 expression had worse prognosis. LINC00501 involved in the progression and development of lung cancer [31]. Our study also showed that LINC00501 is highly expressed in HCC samples. All in vitro experiments suggested that LINC00501 may be a risk factor for prognosis survival. The mechanism of LINC00501 as a risk factor would be our next step to study whether it works as a competing endogenous RNA (ceRNA).

Although this research explored the mechanism of genomic instability in HCC and provided a genomic instability long noncoding RNAs signature, there were still some limitations. We explored the Gene Expression Omnibus database; however, the RNA-seq data and clinical data were inadequate to validate our study. As for molecular biology, only one selected lncRNA was used and further more types of research about the relationship between lncRNAs and genomic instability should be done.

## 5. Conclusions

Our study developed a genomic instability long noncoding RNAs signature to predict HCC patients' overall survival, and we validated the function of LINC00501, one lncRNA of the GIlncSig, with CCK-8, EdU, colony formation, wound healing, and transwell assay.

## Data Availability

The authors confirm that the data supporting the findings of this study are available within the article.

## Supplementary Materials

Supplementary Materials Table S1: The overall clinical characteristics of 353 patients Table S2: The information of HM-group and LM-group Table S3: The information of 52 up-regulated lncRNAs and 83 down-regulated lncRNAs Table S4: The information of GS-group and GU-group Table S5: The information of high-risk group and low-risk group Table S6: The concrete clinical information of 353 patients.
