# Supplementary material for: A Genomic Instability-Related Long Noncoding RNA Signature for Predicting Hepatocellular Carcinoma Prognosis
Source: J Oncol. 2022 Aug 29;2022:3090523. doi: 10.1155/2022/3090523 (PMC9444385; doi:10.1155/2022/3090523)
Supplement: Supplementary Materials — Table S1: The overall clinical characteristics of 353 patients Table S2: The information of HM-group and LM-group Table S3: The information of 52 up-regulated lncRNAs and 83 down-regulated lncRNAs Table S4: The information of GS-group and GU-group Table S5: The information of high-risk group and low-risk group Table S6: The concrete clinical information of 353 patients. [file 3090523.f1.zip › 3090523.f1/TableS1.docx]

| **Variable** | **Total (n=353)** |
| --- | --- |
| **Age (year)** |  |
| <65 | 213 |
| >=65 | 137 |
| NA | 3 |
| **Gender** |  |
| female | 116 |
| male | 237 |
| **Stage** |  |
| Ⅰ | 167 |
| Ⅱ | 80 |
| Ⅲ | 81 |
| Ⅳ | 4 |
| **T stage** |  |
| T1 | 176 |
| T2 | 87 |
| T3 | 74 |
| T4 | 13 |
| **N stage** |  |
| N0 | 240 |
| N1 | 4 |
| Nx | 109 |
| **M stage** |  |
| M0 | 254 |
| M1 | 3 |
| Mx | 96 |
| **Grade** |  |
| G1 | 52 |
| G2 | 171 |
| G3 | 113 |
| G4 | 12 |
| NA | 5 |

TableS1 clinical characteristics of patients
