# Supplementary material for: A Genomic Instability-Related Long Noncoding RNA Signature for Predicting Hepatocellular Carcinoma Prognosis
Source: J Oncol. 2022 Aug 29;2022:3090523. doi: 10.1155/2022/3090523 (PMC9444385; doi:10.1155/2022/3090523)
Supplement: Supplementary Materials — Table S1: The overall clinical characteristics of 353 patients Table S2: The information of HM-group and LM-group Table S3: The information of 52 up-regulated lncRNAs and 83 down-regulated lncRNAs Table S4: The information of GS-group and GU-group Table S5: The information of high-risk group and low-risk group Table S6: The concrete clinical information of 353 patients. [file 3090523.f1.zip › 3090523.f1/TableS2.docx]

| sample | Freq | group |
| --- | --- | --- |
| TCGA.MI.A75C.01A | 156 | HIGH |
| TCGA.ZS.A9CE.01A | 157 | HIGH |
| TCGA.DD.A3A8.01A | 159 | HIGH |
| TCGA.DD.AADB.01A | 159 | HIGH |
| TCGA.G3.AAV4.01A | 159 | HIGH |
| TCGA.CC.5262.01A | 160 | HIGH |
| TCGA.DD.AACX.01A | 161 | HIGH |
| TCGA.CC.5263.01A | 162 | HIGH |
| TCGA.DD.AAD8.01A | 163 | HIGH |
| TCGA.DD.A3A7.01A | 163 | HIGH |
| TCGA.G3.A7M9.01A | 164 | HIGH |
| TCGA.DD.A4NI.01A | 165 | HIGH |
| TCGA.HP.A5N0.01A | 165 | HIGH |
| TCGA.DD.AADL.01A | 166 | HIGH |
| TCGA.2Y.A9H9.01A | 167 | HIGH |
| TCGA.FV.A2QQ.01A | 167 | HIGH |
| TCGA.DD.A113.01A | 168 | HIGH |
| TCGA.G3.A25S.01A | 168 | HIGH |
| TCGA.CC.A7IF.01A | 169 | HIGH |
| TCGA.ED.A8O5.01A | 170 | HIGH |
| TCGA.DD.AADQ.01A | 170 | HIGH |
| TCGA.DD.A73E.01A | 170 | HIGH |
| TCGA.BC.A10U.01A | 171 | HIGH |
| TCGA.CC.5258.01A | 172 | HIGH |
| TCGA.DD.AACF.01A | 173 | HIGH |
| TCGA.DD.A11D.01A | 173 | HIGH |
| TCGA.G3.A6UC.01A | 174 | HIGH |
| TCGA.2Y.A9H3.01A | 174 | HIGH |
| TCGA.RC.A7SK.01A | 174 | HIGH |
| TCGA.DD.AAEB.01A | 176 | HIGH |
| TCGA.MI.A75H.01A | 176 | HIGH |
| TCGA.BC.A69H.01A | 176 | HIGH |
| TCGA.ED.A459.01A | 176 | HIGH |
| TCGA.DD.AAD5.01A | 177 | HIGH |
| TCGA.CC.A5UE.01A | 177 | HIGH |
| TCGA.2Y.A9H1.01A | 178 | HIGH |
| TCGA.2Y.A9GU.01A | 179 | HIGH |
| TCGA.3K.AAZ8.01A | 179 | HIGH |
| TCGA.DD.AAW2.01A | 179 | HIGH |
| TCGA.CC.5259.01A | 181 | HIGH |
| TCGA.ZP.A9D1.01A | 183 | HIGH |
| TCGA.CC.A7IJ.01A | 184 | HIGH |
| TCGA.WJ.A86L.01A | 184 | HIGH |
| TCGA.BC.4073.01B | 184 | HIGH |
| TCGA.DD.A1EB.01A | 187 | HIGH |
| TCGA.DD.AACK.01A | 188 | HIGH |
| TCGA.2Y.A9HA.01A | 188 | HIGH |
| TCGA.DD.AADS.01A | 189 | HIGH |
| TCGA.5C.A9VG.01A | 189 | HIGH |
| TCGA.CC.A8HV.01A | 189 | HIGH |
| TCGA.DD.AADG.01A | 192 | HIGH |
| TCGA.XR.A8TF.01A | 195 | HIGH |
| TCGA.CC.A7IG.01A | 197 | HIGH |
| TCGA.DD.AAE3.01A | 210 | HIGH |
| TCGA.CC.A9FW.01A | 210 | HIGH |
| TCGA.DD.AACZ.01A | 213 | HIGH |
| TCGA.CC.A7II.01A | 219 | HIGH |
| TCGA.CC.A7IE.01A | 223 | HIGH |
| TCGA.DD.AACP.01A | 225 | HIGH |
| TCGA.DD.AADM.01A | 226 | HIGH |
| TCGA.MI.A75I.01A | 231 | HIGH |
| TCGA.G3.AAV0.01A | 236 | HIGH |
| TCGA.DD.AAEA.01A | 240 | HIGH |
| TCGA.RC.A6M4.01A | 241 | HIGH |
| TCGA.BC.A10Z.01A | 243 | HIGH |
| TCGA.RC.A6M6.01A | 245 | HIGH |
| TCGA.G3.A7M5.01A | 252 | HIGH |
| TCGA.LG.A6GG.01A | 263 | HIGH |
| TCGA.CC.A8HT.01A | 267 | HIGH |
| TCGA.DD.AACT.01A | 273 | HIGH |
| TCGA.G3.A3CK.01A | 275 | HIGH |
| TCGA.DD.AADO.01A | 278 | HIGH |
| TCGA.CC.A5UD.01A | 278 | HIGH |
| TCGA.DD.AACQ.01A | 282 | HIGH |
| TCGA.DD.AADF.01A | 295 | HIGH |
| TCGA.MI.A75G.01A | 297 | HIGH |
| TCGA.DD.AAE7.01A | 313 | HIGH |
| TCGA.DD.A1EE.01A | 339 | HIGH |
| TCGA.ED.A7PZ.01A | 341 | HIGH |
| TCGA.CC.A7IK.01A | 400 | HIGH |
| TCGA.DD.AACL.01A | 406 | HIGH |
| TCGA.DD.A3A9.01A | 425 | HIGH |
| TCGA.DD.AACI.01A | 435 | HIGH |
| TCGA.WQ.A9G7.01A | 674 | HIGH |
| TCGA.DD.AAC8.01A | 716 | HIGH |
| TCGA.CC.A7IH.01A | 1040 | HIGH |
| TCGA.4R.AA8I.01A | 1559 | HIGH |
| TCGA.UB.A7MB.01A | 2055 | HIGH |
| TCGA.ED.A627.01A | 3 | LOW |
| TCGA.ED.A5KG.01A | 4 | LOW |
| TCGA.BC.A10X.01A | 5 | LOW |
| TCGA.K7.A5RF.01A | 10 | LOW |
| TCGA.5R.AA1D.01A | 15 | LOW |
| TCGA.RC.A6M5.01A | 16 | LOW |
| TCGA.DD.A3A6.01A | 16 | LOW |
| TCGA.G3.A3CI.01A | 19 | LOW |
| TCGA.DD.A4NL.01A | 19 | LOW |
| TCGA.XR.A8TE.01A | 19 | LOW |
| TCGA.DD.A4NS.01A | 21 | LOW |
| TCGA.G3.A7M8.01A | 24 | LOW |
| TCGA.MR.A520.01A | 24 | LOW |
| TCGA.DD.A4NP.01A | 26 | LOW |
| TCGA.DD.A1ED.01A | 28 | LOW |
| TCGA.MR.A8JO.01A | 29 | LOW |
| TCGA.DD.A4NB.01A | 33 | LOW |
| TCGA.ED.A7PY.01A | 36 | LOW |
| TCGA.DD.A1EC.01A | 36 | LOW |
| TCGA.DD.A4NA.01A | 39 | LOW |
| TCGA.DD.A1EH.01A | 40 | LOW |
| TCGA.DD.AAVW.01A | 41 | LOW |
| TCGA.ED.A82E.01A | 42 | LOW |
| TCGA.G3.A3CH.01A | 43 | LOW |
| TCGA.WX.AA46.01A | 43 | LOW |
| TCGA.ED.A7PX.01A | 43 | LOW |
| TCGA.UB.AA0V.01A | 44 | LOW |
| TCGA.G3.A5SI.01A | 45 | LOW |
| TCGA.DD.A39W.01A | 46 | LOW |
| TCGA.FV.A3R3.01A | 48 | LOW |
| TCGA.DD.A3A4.01A | 48 | LOW |
| TCGA.ED.A66X.01A | 51 | LOW |
| TCGA.WX.AA47.01A | 51 | LOW |
| TCGA.2Y.A9GX.01A | 52 | LOW |
| TCGA.KR.A7K8.01A | 53 | LOW |
| TCGA.GJ.A3OU.01A | 54 | LOW |
| TCGA.BC.A10Q.01A | 54 | LOW |
| TCGA.DD.AACN.01A | 56 | LOW |
| TCGA.DD.AADW.01A | 56 | LOW |
| TCGA.HP.A5MZ.01A | 56 | LOW |
| TCGA.PD.A5DF.01A | 56 | LOW |
| TCGA.DD.AA3A.01A | 57 | LOW |
| TCGA.DD.AAVQ.01A | 62 | LOW |
| TCGA.DD.AAVZ.01A | 62 | LOW |
| TCGA.CC.A3MA.01A | 62 | LOW |
| TCGA.CC.A8HS.01A | 63 | LOW |
| TCGA.DD.A4NH.01A | 63 | LOW |
| TCGA.DD.AACW.01A | 64 | LOW |
| TCGA.DD.AADY.01A | 64 | LOW |
| TCGA.DD.AAVS.01A | 65 | LOW |
| TCGA.EP.A12J.01A | 65 | LOW |
| TCGA.DD.A4NO.01A | 65 | LOW |
| TCGA.DD.AACS.01A | 65 | LOW |
| TCGA.ED.A97K.01A | 65 | LOW |
| TCGA.DD.AACO.01A | 66 | LOW |
| TCGA.DD.A4ND.01A | 66 | LOW |
| TCGA.LG.A9QC.01A | 66 | LOW |
| TCGA.XR.A8TC.01A | 67 | LOW |
| TCGA.DD.A1EI.01A | 67 | LOW |
| TCGA.CC.5260.01A | 67 | LOW |
| TCGA.DD.A4NR.01A | 67 | LOW |
| TCGA.DD.AAE1.01A | 68 | LOW |
| TCGA.BD.A3ER.01A | 68 | LOW |
| TCGA.G3.A25T.01A | 68 | LOW |
| TCGA.DD.AADK.01A | 69 | LOW |
| TCGA.ED.A7XO.01A | 70 | LOW |
| TCGA.G3.A25V.01A | 70 | LOW |
| TCGA.ZP.A9CZ.01A | 71 | LOW |
| TCGA.CC.A5UC.01A | 71 | LOW |
| TCGA.XR.A8TD.01A | 71 | LOW |
| TCGA.EP.A3RK.01A | 71 | LOW |
| TCGA.DD.A119.01A | 71 | LOW |
| TCGA.2Y.A9H2.01A | 72 | LOW |
| TCGA.DD.A3A3.01A | 73 | LOW |
| TCGA.G3.AAV2.01A | 73 | LOW |
| TCGA.DD.A39X.01A | 73 | LOW |
| TCGA.DD.A4NN.01A | 74 | LOW |
| TCGA.DD.A11B.01A | 75 | LOW |
| TCGA.ZP.A9D0.01A | 75 | LOW |
| TCGA.2Y.A9H6.01A | 76 | LOW |
| TCGA.KR.A7K2.01A | 76 | LOW |
| TCGA.RC.A7S9.01A | 76 | LOW |
| TCGA.G3.AAV1.01A | 76 | LOW |
| TCGA.DD.A1EK.01A | 77 | LOW |
| TCGA.DD.A1EJ.01A | 77 | LOW |
| TCGA.2Y.A9GV.01A | 78 | LOW |
| TCGA.DD.A4NE.01A | 78 | LOW |
| TCGA.KR.A7K7.01A | 78 | LOW |
