# Supplementary material for: A Genomic Instability-Related Long Noncoding RNA Signature for Predicting Hepatocellular Carcinoma Prognosis
Source: J Oncol. 2022 Aug 29;2022:3090523. doi: 10.1155/2022/3090523 (PMC9444385; doi:10.1155/2022/3090523)
Supplement: Supplementary Materials — Table S1: The overall clinical characteristics of 353 patients Table S2: The information of HM-group and LM-group Table S3: The information of 52 up-regulated lncRNAs and 83 down-regulated lncRNAs Table S4: The information of GS-group and GU-group Table S5: The information of high-risk group and low-risk group Table S6: The concrete clinical information of 353 patients. [file 3090523.f1.zip › 3090523.f1/TableS3.docx]

|  | logFC | AveExpr | t | P.Value | adj.P.Val | B | change |
| --- | --- | --- | --- | --- | --- | --- | --- |
| CTD-3157E16.1 | -1.6960391 | 2.61342275 | -7.6647089 | 1.19E-12 | 6.29E-09 | 18.1670969 | DOWN |
| RP5-1180D12.1 | -1.700044 | 4.06583931 | -6.8800521 | 1.03E-10 | 2.76E-07 | 13.9626204 | DOWN |
| CTD-2536I1.2 | -1.3548107 | 2.07787377 | -6.8770346 | 1.04E-10 | 2.76E-07 | 13.9469178 | DOWN |
| RP11-588K22.2 | -1.4200999 | 7.12872784 | -6.4741404 | 9.32E-10 | 1.64E-06 | 11.8858646 | DOWN |
| AP000892.6 | -1.3133011 | 5.14969413 | -6.1419854 | 5.35E-09 | 7.06E-06 | 10.2428848 | DOWN |
| RP5-1007F24.1 | -1.262243 | 1.18317773 | -6.0812276 | 7.31E-09 | 8.58E-06 | 9.9481878 | DOWN |
| RP11-672A2.4 | -1.4655391 | 2.95478894 | -6.0524781 | 8.48E-09 | 8.96E-06 | 9.80939178 | DOWN |
| CTB-41I6.2 | -1.0439937 | 1.78353179 | -5.940724 | 1.50E-08 | 1.44E-05 | 9.2738845 | DOWN |
| AP001189.4 | -1.3457627 | 2.5387174 | -5.9045686 | 1.80E-08 | 1.55E-05 | 9.10201934 | DOWN |
| UNC5C-AS1 | -1.1298868 | 1.98454555 | -5.8937481 | 1.90E-08 | 1.55E-05 | 9.05071719 | DOWN |
| SH3RF3-AS1 | -1.6189947 | 3.37460914 | -5.8485727 | 2.39E-08 | 1.76E-05 | 8.83719865 | DOWN |
| RP1-47M23.3 | -1.4191279 | 2.98821895 | -5.8394274 | 2.50E-08 | 1.76E-05 | 8.7941053 | DOWN |
| LINC01679 | -1.079111 | 3.94036002 | -5.8102366 | 2.89E-08 | 1.91E-05 | 8.6568549 | DOWN |
| AF131215.9 | -1.2730162 | 3.86366188 | -5.7699616 | 3.54E-08 | 2.20E-05 | 8.46823773 | DOWN |
| AF131215.2 | -1.6457769 | 2.88903775 | -5.7532151 | 3.85E-08 | 2.26E-05 | 8.3900675 | DOWN |
| AC000403.4 | -0.9906416 | 2.5978522 | -5.6506708 | 6.39E-08 | 3.48E-05 | 7.91473621 | DOWN |
| RP11-454H19.2 | -0.9744992 | 1.03612278 | -5.5736757 | 9.31E-08 | 4.68E-05 | 7.56165072 | DOWN |
| CTD-2334D19.1 | -0.9553346 | 1.28624988 | -5.5381705 | 1.11E-07 | 5.31E-05 | 7.39994823 | DOWN |
| CTD-3157E16.2 | -0.9898032 | 4.22850206 | -5.4801494 | 1.47E-07 | 6.45E-05 | 7.13723789 | DOWN |
| RP11-88E10.4 | -1.2324679 | 2.99350639 | -5.4620295 | 1.60E-07 | 6.50E-05 | 7.05558708 | DOWN |
| RP11-284N8.3 | -1.2978622 | 4.26204658 | -5.4517215 | 1.68E-07 | 6.57E-05 | 7.0092217 | DOWN |
| RP11-92C4.6 | -1.0336879 | 1.97164454 | -5.3793697 | 2.38E-07 | 8.09E-05 | 6.68551102 | DOWN |
| RP11-59C5.3 | -1.1021456 | 3.92966318 | -5.3538096 | 2.68E-07 | 8.85E-05 | 6.57187917 | DOWN |
| EPB41L4A-DT | -1.0268252 | 3.99906682 | -5.2858285 | 3.70E-07 | 0.00010854 | 6.27152508 | DOWN |
| RP11-4B16.3 | -0.9174197 | 1.27216161 | -5.1767442 | 6.16E-07 | 0.00016686 | 5.79531299 | DOWN |
| RP11-532F6.3 | -0.9800952 | 3.37207493 | -5.1376762 | 7.38E-07 | 0.00019495 | 5.62650433 | DOWN |
| AC012360.6 | -0.9222175 | 1.38058882 | -5.1256799 | 7.80E-07 | 0.00019624 | 5.57485612 | DOWN |
| CTD-3064M3.3 | -1.3220384 | 3.83698517 | -5.0463438 | 1.12E-06 | 0.00025786 | 5.2355093 | DOWN |
| AGAP2-AS1 | -1.0278188 | 5.99020661 | -5.0362677 | 1.18E-06 | 0.00026424 | 5.19268848 | DOWN |
| RP1-18D14.7 | -0.987433 | 2.27459702 | -5.0135892 | 1.30E-06 | 0.00027582 | 5.09654113 | DOWN |
| RP11-800A3.7 | -1.0015308 | 2.6335833 | -5.0084915 | 1.33E-06 | 0.00027582 | 5.07497333 | DOWN |
| RP11-13P5.2 | -0.9056307 | 1.06652569 | -5.000411 | 1.38E-06 | 0.00027582 | 5.04081878 | DOWN |
| LINC00920 | -0.973315 | 3.84393215 | -4.9441637 | 1.78E-06 | 0.00031946 | 4.80420575 | DOWN |
| RP11-34A14.3 | -1.1686712 | 1.49771406 | -4.9074856 | 2.10E-06 | 0.00036429 | 4.65098744 | DOWN |
| AC025165.8 | -1.108698 | 1.90912475 | -4.8916302 | 2.26E-06 | 0.00037865 | 4.58501769 | DOWN |
| RP11-473M20.5 | -1.2754333 | 1.84038669 | -4.8501765 | 2.72E-06 | 0.00042196 | 4.41329798 | DOWN |
| RP11-355F16.1 | -1.0425468 | 2.25987416 | -4.8006734 | 3.38E-06 | 0.0005016 | 4.20967849 | DOWN |
| RP11-573G6.4 | -1.0164329 | 2.09386271 | -4.7981888 | 3.42E-06 | 0.0005016 | 4.19950052 | DOWN |
| CTC-455F18.1 | -0.980475 | 1.44269205 | -4.77314 | 3.82E-06 | 0.00053765 | 4.09711119 | DOWN |
| RP11-863P13.4 | -0.9223999 | 1.3510979 | -4.7254419 | 4.70E-06 | 0.00064527 | 3.90326953 | DOWN |
| RP11-266K4.14 | -0.9925983 | 2.21362168 | -4.6722828 | 5.93E-06 | 0.00074608 | 3.68899123 | DOWN |
| RP11-1134I14.8 | -1.1705354 | 2.53637682 | -4.6682863 | 6.03E-06 | 0.00074608 | 3.6729567 | DOWN |
| RP11-789C17.1 | -1.0103923 | 2.45399867 | -4.6286667 | 7.15E-06 | 0.00083022 | 3.51457278 | DOWN |
| AC106786.1 | -0.9758825 | 1.53984442 | -4.5543476 | 9.83E-06 | 0.00105918 | 3.22029582 | DOWN |
| ERVE-1 | -1.2018098 | 1.05222165 | -4.5153899 | 1.16E-05 | 0.00118863 | 3.06751999 | DOWN |
| LINC02688 | -1.5160727 | 2.49234693 | -4.4648487 | 1.43E-05 | 0.00139256 | 2.8708522 | DOWN |
| RP11-728F11.3 | -1.4703618 | 1.95601457 | -4.4627224 | 1.45E-05 | 0.00139256 | 2.86261644 | DOWN |
| RP11-510M2.5 | -1.5178929 | 1.55922351 | -4.2929384 | 2.92E-05 | 0.00237028 | 2.21505037 | DOWN |
| RP11-747H7.3 | -1.3794766 | 4.35397808 | -4.2742961 | 3.15E-05 | 0.00246757 | 2.14516993 | DOWN |
| RP11-7K24.3 | -1.2233398 | 2.05786964 | -4.2552717 | 3.40E-05 | 0.00254972 | 2.07410925 | DOWN |
| CTD-2541J13.1 | -1.1061897 | 2.38329895 | -4.2072334 | 4.13E-05 | 0.00294451 | 1.89581201 | DOWN |
| RP4-568C11.4 | -1.6909136 | 5.56793171 | -4.180462 | 4.59E-05 | 0.00319154 | 1.79715875 | DOWN |
| ADIRF-AS1 | -0.9582352 | 4.29323355 | -4.1524956 | 5.14E-05 | 0.00347645 | 1.69464784 | DOWN |
| AC144831.1 | -0.9702789 | 3.38070565 | -4.0144823 | 8.83E-05 | 0.00532927 | 1.19700467 | DOWN |
| RP11-43F13.3 | -1.460515 | 3.37373824 | -3.9760992 | 0.00010243 | 0.00588347 | 1.06106534 | DOWN |
| CH17-360D5.3 | -1.1935938 | 1.63537195 | -3.9564512 | 0.00011047 | 0.00610833 | 0.99189729 | DOWN |
| LINC02894 | -1.1994411 | 1.31534085 | -3.9443004 | 0.00011574 | 0.00626671 | 0.94926449 | DOWN |
| CTD-3099C6.11 | -0.9603455 | 3.47977918 | -3.937897 | 0.00011861 | 0.00628767 | 0.92684085 | DOWN |
| RP11-465B22.8 | -1.2468152 | 2.45942626 | -3.8993665 | 0.00013737 | 0.00684328 | 0.79255434 | DOWN |
| LINC01857 | -0.9610235 | 2.63221604 | -3.8836136 | 0.00014583 | 0.00716274 | 0.73796892 | DOWN |
| RP13-580F15.2 | -0.9366381 | 3.04995151 | -3.8715495 | 0.00015263 | 0.00732704 | 0.69629073 | DOWN |
| CTD-2600O9.1 | -0.9653843 | 3.84651066 | -3.8405578 | 0.00017153 | 0.00787608 | 0.58971998 | DOWN |
| AC016735.1 | -1.2703561 | 2.71417294 | -3.7958647 | 0.00020272 | 0.00873846 | 0.43730042 | DOWN |
| RP11-253E3.3 | -0.9597951 | 4.3760511 | -3.7352745 | 0.00025366 | 0.00992205 | 0.2330679 | DOWN |
| RP11-701H24.3 | -0.9072031 | 1.84117802 | -3.7317283 | 0.00025699 | 0.00994867 | 0.22120075 | DOWN |
| HAR1A | -0.9337842 | 2.35079461 | -3.7266559 | 0.00026183 | 0.01001869 | 0.20424298 | DOWN |
| RP11-172H24.4 | -0.9450094 | 2.35017032 | -3.7139455 | 0.00027432 | 0.01023488 | 0.16183546 | DOWN |
| RP11-404P21.3 | -1.4744048 | 4.93746843 | -3.7132762 | 0.00027499 | 0.01023488 | 0.15960593 | DOWN |
| LINC02593 | -1.2508599 | 3.45835604 | -3.6976296 | 0.00029118 | 0.0106357 | 0.10757923 | DOWN |
| LINC02600 | -1.2687486 | 3.41979997 | -3.6075897 | 0.00040328 | 0.01355711 | -0.1881754 | DOWN |
| PRKAR1B-AS2 | -1.3377578 | 1.52478649 | -3.6068411 | 0.00040436 | 0.01355711 | -0.1906081 | DOWN |
| RP11-503C24.6 | -1.100154 | 4.75015259 | -3.5471304 | 0.00050017 | 0.01550938 | -0.3832621 | DOWN |
| LINC02038 | -0.927088 | 2.37331458 | -3.3839033 | 0.00088233 | 0.02300812 | -0.8957057 | DOWN |
| HECW2-AS1 | -0.9049359 | 2.20879827 | -3.322781 | 0.00108565 | 0.02678867 | -1.0821829 | DOWN |
| PWAR5 | -0.9769743 | 2.91704548 | -3.3124024 | 0.00112424 | 0.0272945 | -1.1135512 | DOWN |
| LINC01979 | -0.9240304 | 3.18472426 | -3.2699681 | 0.00129571 | 0.02942805 | -1.2409073 | DOWN |
| SLCO4A1-AS1 | -0.9197734 | 1.58962287 | -3.1433824 | 0.00196269 | 0.03852776 | -1.6122014 | DOWN |
| KRT7-AS | -1.0190121 | 2.87455802 | -3.0606053 | 0.00255783 | 0.04545965 | -1.8479482 | DOWN |
| LINC00239 | -0.9008988 | 2.96352391 | -2.929351 | 0.00385031 | 0.05883608 | -2.2101852 | DOWN |
| RP11-156K13.1 | -0.9083263 | 6.6093095 | -2.7851838 | 0.0059404 | 0.07604436 | -2.5914975 | DOWN |
| LY6E-DT | -0.9050975 | 3.78776864 | -2.6347884 | 0.00917577 | 0.10104829 | -2.9705398 | DOWN |
| RP3-523K23.2 | -0.9092625 | 1.91027076 | -2.2566669 | 0.02526843 | 0.18215689 | -3.8371729 | DOWN |
| RP11-25H12.1 | 2.3490972 | 2.45422056 | 8.41574798 | 1.36E-14 | 1.44E-10 | 22.388293 | UP |
| RP11-313L6.2 | 0.95995994 | 0.59747829 | 6.5623928 | 5.81E-10 | 1.23E-06 | 12.3311478 | UP |
| CTD-2591A6.2 | 1.80724416 | 1.50293919 | 6.4276564 | 1.19E-09 | 1.80E-06 | 11.6527715 | UP |
| RP11-308B16.2 | 1.40797403 | 1.18191246 | 5.48609021 | 1.42E-07 | 6.45E-05 | 7.16404876 | UP |
| CTD-2374C24.1 | 1.5122828 | 1.49985739 | 5.46927555 | 1.54E-07 | 6.50E-05 | 7.0882162 | UP |
| RP1-140A9.1 | 1.11578734 | 3.73357391 | 5.41040292 | 2.05E-07 | 7.46E-05 | 6.82398608 | UP |
| RP11-21L23.3 | 1.27081457 | 3.99094851 | 5.38018245 | 2.37E-07 | 8.09E-05 | 6.68913034 | UP |
| RP11-84N19.1 | 1.33124242 | 1.2313662 | 5.33213875 | 2.97E-07 | 9.36E-05 | 6.47583731 | UP |
| CTA-280A3.2 | 1.04498345 | 0.90183777 | 5.31193922 | 3.27E-07 | 9.87E-05 | 6.38656491 | UP |
| CDKN2A-DT | 0.94202425 | 1.39857436 | 5.26134834 | 4.15E-07 | 0.00011849 | 6.1640363 | UP |
| RP11-278J6.4 | 0.9006832 | 3.39463983 | 5.18412301 | 5.95E-07 | 0.00016548 | 5.82729999 | UP |
| LINC01419 | 2.96964669 | 3.41470318 | 5.08069901 | 9.60E-07 | 0.00023037 | 5.3819823 | UP |
| RP11-109M17.2 | 1.70609406 | 1.73469123 | 5.05407041 | 1.08E-06 | 0.00025445 | 5.26838793 | UP |
| RP4-533D7.5 | 0.94434411 | 1.74961651 | 5.02967722 | 1.21E-06 | 0.00026662 | 5.16471459 | UP |
| RP11-42O4.2 | 0.9049628 | 2.17193135 | 4.98359311 | 1.49E-06 | 0.00028683 | 4.96986353 | UP |
| RP11-52L5.6 | 1.01931231 | 0.92906307 | 4.96736017 | 1.61E-06 | 0.00030316 | 4.90154439 | UP |
| CTC-480C2.1 | 1.33559413 | 1.23805629 | 4.85095311 | 2.71E-06 | 0.00042196 | 4.41650496 | UP |
| DHX35-DT | 0.93552991 | 3.17897805 | 4.82533287 | 3.03E-06 | 0.00046426 | 4.31091225 | UP |
| RP11-96B2.1 | 1.35294726 | 2.29078666 | 4.80798992 | 3.27E-06 | 0.00049409 | 4.23967383 | UP |
| RP1-209A6.1 | 1.04974117 | 1.20755064 | 4.66656026 | 6.08E-06 | 0.00074608 | 3.66603515 | UP |
| LINC02335 | 1.3741703 | 1.41056197 | 4.53686342 | 1.06E-05 | 0.00112924 | 3.15160333 | UP |
| CASC20 | 1.3711446 | 1.24612822 | 4.52052869 | 1.13E-05 | 0.0011745 | 3.08761346 | UP |
| AC007128.1 | 1.10195869 | 1.09732906 | 4.38337153 | 2.01E-05 | 0.00181819 | 2.55748447 | UP |
| LINC00501 | 1.03865332 | 1.31352615 | 4.32301128 | 2.58E-05 | 0.00215082 | 2.32829176 | UP |
| LINC02820 | 1.13428243 | 1.04082908 | 4.32118623 | 2.60E-05 | 0.00215082 | 2.32140141 | UP |
| LINC02819 | 1.30815649 | 2.51255387 | 4.27180683 | 3.18E-05 | 0.00246757 | 2.13585742 | UP |
| LL22NC03-N14H11.1 | 0.993152 | 2.06201478 | 4.14495503 | 5.29E-05 | 0.00355938 | 1.66710361 | UP |
| AC016710.1 | 1.31496538 | 1.24525979 | 4.03836204 | 8.05E-05 | 0.00497361 | 1.28212174 | UP |
| RP11-1041F24.1 | 1.40588051 | 1.37537346 | 4.02603648 | 8.44E-05 | 0.00512471 | 1.23813662 | UP |
| RP11-91I8.2 | 0.91565603 | 0.93548931 | 4.01198936 | 8.92E-05 | 0.00534615 | 1.18814295 | UP |
| SMILR | 1.02129958 | 1.98389853 | 3.85911171 | 0.00015996 | 0.00750839 | 0.6534349 | UP |
| LINC01124 | 1.22207615 | 7.13865106 | 3.82924246 | 0.00017896 | 0.0080086 | 0.55098885 | UP |
| RP11-320H14.1 | 1.12662191 | 0.91560895 | 3.82375443 | 0.00018268 | 0.00807227 | 0.53223854 | UP |
| LINC02562 | 1.03329548 | 1.3124052 | 3.78837285 | 0.00020845 | 0.00884574 | 0.41189723 | UP |
| LINC01793 | 1.06605356 | 0.95587641 | 3.71659526 | 0.00027167 | 0.01023488 | 0.17066619 | UP |
| RP11-47P18.1 | 1.06254215 | 1.18831252 | 3.71208067 | 0.0002762 | 0.01023488 | 0.15562407 | UP |
| RP11-283I3.4 | 1.16270768 | 3.30844448 | 3.5212192 | 0.00054806 | 0.01635053 | -0.4660023 | UP |
| DCXR-DT | 1.1135931 | 5.8604489 | 3.44576557 | 0.00071323 | 0.01992702 | -0.7039553 | UP |
| LINC01639 | 1.14398881 | 2.30400571 | 3.34571356 | 0.00100472 | 0.02538482 | -1.0125668 | UP |
| RP11-1038A11.3 | 1.05681161 | 1.36990944 | 3.28329818 | 0.00123939 | 0.02841425 | -1.2010559 | UP |
| XX-C2158C6.3 | 0.92443197 | 1.79161276 | 3.16059636 | 0.00185627 | 0.03719945 | -1.5624735 | UP |
| RP11-575F12.3 | 1.10999895 | 5.89426066 | 3.14824903 | 0.00193204 | 0.03806201 | -1.5981671 | UP |
| SYN3-AS1 | 0.98779897 | 3.3146211 | 3.1346465 | 0.00201882 | 0.03919266 | -1.6373455 | UP |
| RP11-21L23.2 | 1.00718549 | 7.65045379 | 3.10812999 | 0.00219845 | 0.04138657 | -1.7132849 | UP |
| AP000593.7 | 1.03988033 | 2.28231378 | 3.09620372 | 0.00228397 | 0.04261656 | -1.7472525 | UP |
| CTD-2280E9.1 | 0.90670734 | 2.50035243 | 3.09197896 | 0.00231499 | 0.04301359 | -1.7592573 | UP |
| RP11-669E14.4 | 1.02266808 | 3.59862089 | 3.02519282 | 0.00286003 | 0.04879608 | -1.9470837 | UP |
| RP1-170O19.14 | 0.94877788 | 2.13787382 | 2.96727441 | 0.00342591 | 0.05448952 | -2.1069907 | UP |
| RP5-1154L15.2 | 1.27439349 | 4.4034668 | 2.91270455 | 0.00405139 | 0.06064818 | -2.2551037 | UP |
| RP11-60A14.1 | 0.92598615 | 2.78484296 | 2.90351972 | 0.0041664 | 0.06154042 | -2.2797889 | UP |
| RP11-76C10.6 | 1.09350848 | 1.47542072 | 2.89577866 | 0.00426566 | 0.06256888 | -2.300539 | UP |
| RP11-238F2.1 | 1.36041507 | 3.03631576 | 2.74451348 | 0.00669345 | 0.08142155 | -2.6958986 | UP |
| RP11-115C10.1 | 1.09613488 | 4.5936428 | 2.21375441 | 0.02814073 | 0.19437162 | -3.9275884 | UP |
