# Supplementary material for: A Genomic Instability-Related Long Noncoding RNA Signature for Predicting Hepatocellular Carcinoma Prognosis
Source: J Oncol. 2022 Aug 29;2022:3090523. doi: 10.1155/2022/3090523 (PMC9444385; doi:10.1155/2022/3090523)
Supplement: Supplementary Materials — Table S1: The overall clinical characteristics of 353 patients Table S2: The information of HM-group and LM-group Table S3: The information of 52 up-regulated lncRNAs and 83 down-regulated lncRNAs Table S4: The information of GS-group and GU-group Table S5: The information of high-risk group and low-risk group Table S6: The concrete clinical information of 353 patients. [file 3090523.f1.zip › 3090523.f1/TableS4.docx]

|  | group |
| --- | --- |
| TCGA.ED.A627.01A | GS_group |
| TCGA.ED.A5KG.01A | GS_group |
| TCGA.BC.A10X.01A | GS_group |
| TCGA.K7.A5RF.01A | GS_group |
| TCGA.5R.AA1D.01A | GS_group |
| TCGA.RC.A6M5.01A | GS_group |
| TCGA.DD.A3A6.01A | GS_group |
| TCGA.G3.A3CI.01A | GS_group |
| TCGA.DD.A4NL.01A | GS_group |
| TCGA.DD.A4NS.01A | GS_group |
| TCGA.G3.A7M8.01A | GS_group |
| TCGA.MR.A520.01A | GS_group |
| TCGA.DD.A1ED.01A | GS_group |
| TCGA.MR.A8JO.01A | GS_group |
| TCGA.DD.A4NB.01A | GS_group |
| TCGA.ED.A7PY.01A | GS_group |
| TCGA.DD.A1EC.01A | GS_group |
| TCGA.DD.A4NA.01A | GS_group |
| TCGA.DD.A1EH.01A | GS_group |
| TCGA.DD.AAVW.01A | GS_group |
| TCGA.ED.A82E.01A | GS_group |
| TCGA.G3.A3CH.01A | GS_group |
| TCGA.WX.AA46.01A | GS_group |
| TCGA.ED.A7PX.01A | GS_group |
| TCGA.UB.AA0V.01A | GS_group |
| TCGA.FV.A3R3.01A | GS_group |
| TCGA.DD.A3A4.01A | GS_group |
| TCGA.ED.A66X.01A | GS_group |
| TCGA.WX.AA47.01A | GS_group |
| TCGA.2Y.A9GX.01A | GS_group |
| TCGA.GJ.A3OU.01A | GS_group |
| TCGA.BC.A10Q.01A | GS_group |
| TCGA.DD.AACN.01A | GS_group |
| TCGA.DD.AADW.01A | GS_group |
| TCGA.HP.A5MZ.01A | GS_group |
| TCGA.PD.A5DF.01A | GS_group |
| TCGA.DD.AA3A.01A | GS_group |
| TCGA.DD.AAVQ.01A | GS_group |
| TCGA.DD.AAVZ.01A | GS_group |
| TCGA.CC.A3MA.01A | GS_group |
| TCGA.CC.A8HS.01A | GS_group |
| TCGA.DD.A4NH.01A | GS_group |
| TCGA.DD.AACW.01A | GS_group |
| TCGA.DD.AADY.01A | GS_group |
| TCGA.DD.AAVS.01A | GS_group |
| TCGA.DD.A4NO.01A | GS_group |
| TCGA.ED.A97K.01A | GS_group |
| TCGA.DD.A4ND.01A | GS_group |
| TCGA.XR.A8TC.01A | GS_group |
| TCGA.DD.A1EI.01A | GS_group |
| TCGA.CC.5260.01A | GS_group |
| TCGA.DD.A4NR.01A | GS_group |
| TCGA.BD.A3ER.01A | GS_group |
| TCGA.G3.A25T.01A | GS_group |
| TCGA.DD.AADK.01A | GS_group |
| TCGA.ED.A7XO.01A | GS_group |
| TCGA.G3.A25V.01A | GS_group |
| TCGA.ZP.A9CZ.01A | GS_group |
| TCGA.CC.A5UC.01A | GS_group |
| TCGA.EP.A3RK.01A | GS_group |
| TCGA.DD.A119.01A | GS_group |
| TCGA.2Y.A9H2.01A | GS_group |
| TCGA.DD.A39X.01A | GS_group |
| TCGA.DD.A4NN.01A | GS_group |
| TCGA.DD.A11B.01A | GS_group |
| TCGA.ZP.A9D0.01A | GS_group |
| TCGA.2Y.A9H6.01A | GS_group |
| TCGA.RC.A7S9.01A | GS_group |
| TCGA.DD.A1EK.01A | GS_group |
| TCGA.2Y.A9GV.01A | GS_group |
| TCGA.DD.A4NE.01A | GS_group |
| TCGA.G3.A25Y.01A | GS_group |
| TCGA.BC.A216.01A | GS_group |
| TCGA.ED.A7XP.01A | GS_group |
| TCGA.ZP.A9D2.01A | GS_group |
| TCGA.CC.A3MB.01A | GS_group |
| TCGA.2Y.A9GS.01A | GS_group |
| TCGA.RC.A7SB.01A | GS_group |
| TCGA.DD.A73A.01A | GS_group |
| TCGA.G3.A25Z.01A | GS_group |
| TCGA.5R.AAAM.01A | GS_group |
| TCGA.RC.A7SH.01A | GS_group |
| TCGA.DD.AAD3.01A | GS_group |
| TCGA.DD.AAE0.01A | GS_group |
| TCGA.DD.AACC.01A | GS_group |
| TCGA.DD.AAC9.01A | GS_group |
| TCGA.DD.A73F.01A | GS_group |
| TCGA.ED.A8O6.01A | GS_group |
| TCGA.DD.AAVR.01A | GS_group |
| TCGA.K7.AAU7.01A | GS_group |
| TCGA.DD.A4NJ.01A | GS_group |
| TCGA.DD.A4NG.01A | GS_group |
| TCGA.FV.A3I1.01A | GS_group |
| TCGA.YA.A8S7.01A | GS_group |
| TCGA.ES.A2HT.01A | GS_group |
| TCGA.DD.AAD2.01A | GS_group |
| TCGA.2Y.A9GW.01A | GS_group |
| TCGA.2Y.A9HB.01A | GS_group |
| TCGA.BC.A8YO.01A | GS_group |
| TCGA.DD.A73C.01A | GS_group |
| TCGA.BD.A2L6.01A | GS_group |
| TCGA.WX.AA44.01A | GS_group |
| TCGA.BC.A10T.01A | GS_group |
| TCGA.2Y.A9H8.01A | GS_group |
| TCGA.2Y.A9H5.01A | GS_group |
| TCGA.ZP.A9CY.01A | GS_group |
| TCGA.2Y.A9H7.01A | GS_group |
| TCGA.DD.AAVV.01A | GS_group |
| TCGA.FV.A3I0.01A | GS_group |
| TCGA.ED.A4XI.01A | GS_group |
| TCGA.CC.A1HT.01A | GS_group |
| TCGA.O8.A75V.01A | GS_group |
| TCGA.XR.A8TG.01A | GS_group |
| TCGA.EP.A2KC.01A | GS_group |
| TCGA.UB.AA0U.01A | GS_group |
| TCGA.EP.A3JL.01A | GS_group |
| TCGA.DD.A115.01A | GS_group |
| TCGA.FV.A23B.01A | GS_group |
| TCGA.DD.AADV.01A | GS_group |
| TCGA.UB.A7ME.01A | GS_group |
| TCGA.2Y.A9GT.01A | GS_group |
| TCGA.ED.A66Y.01A | GS_group |
| TCGA.DD.AAW0.01A | GS_group |
| TCGA.DD.AADI.01A | GS_group |
| TCGA.G3.AAV7.01A | GS_group |
| TCGA.UB.A7MA.01A | GS_group |
| TCGA.EP.A2KB.01A | GS_group |
| TCGA.GJ.A9DB.01A | GS_group |
| TCGA.T1.A6J8.01A | GS_group |
| TCGA.DD.AAEG.01A | GS_group |
| TCGA.BD.A3EP.01A | GS_group |
| TCGA.ZS.A9CD.01A | GS_group |
| TCGA.CC.A3MC.01A | GS_group |
| TCGA.G3.A7M6.01A | GS_group |
| TCGA.DD.A11C.01A | GS_group |
| TCGA.DD.A114.01A | GS_group |
| TCGA.BC.A10R.01A | GS_group |
| TCGA.DD.AAE2.01A | GS_group |
| TCGA.QA.A7B7.01A | GS_group |
| TCGA.DD.AADP.01A | GS_group |
| TCGA.2Y.A9GZ.01A | GS_group |
| TCGA.DD.AAEH.01A | GS_group |
| TCGA.UB.A7MF.01A | GS_group |
| TCGA.ZS.A9CF.01A | GS_group |
| TCGA.CC.A9FS.01A | GS_group |
| TCGA.CC.5264.01A | GS_group |
| TCGA.DD.A4NV.01A | GS_group |
| TCGA.G3.A5SJ.01A | GS_group |
| TCGA.BC.A10W.01A | GS_group |
| TCGA.DD.A11A.01A | GS_group |
| TCGA.DD.A73G.01A | GS_group |
| TCGA.5C.A9VH.01A | GS_group |
| TCGA.DD.A1EF.01A | GS_group |
| TCGA.K7.A5RG.01A | GS_group |
| TCGA.NI.A8LF.01A | GS_group |
| TCGA.DD.AACH.01A | GS_group |
| TCGA.DD.AACU.01A | GS_group |
| TCGA.DD.AAD1.01A | GS_group |
| TCGA.UB.A7MD.01A | GS_group |
| TCGA.EP.A2KA.01A | GS_group |
| TCGA.MI.A75E.01A | GS_group |
| TCGA.FV.A2QR.01A | GS_group |
| TCGA.G3.A5SM.01A | GS_group |
| TCGA.DD.AADA.01A | GS_group |
| TCGA.CC.A3M9.01A | GS_group |
| TCGA.DD.A118.01A | GS_group |
| TCGA.DD.AADB.01A | GS_group |
| TCGA.CC.5262.01A | GS_group |
| TCGA.CC.5263.01A | GS_group |
| TCGA.G3.A7M9.01A | GS_group |
| TCGA.DD.A4NI.01A | GS_group |
| TCGA.HP.A5N0.01A | GS_group |
| TCGA.DD.AADL.01A | GS_group |
| TCGA.2Y.A9H9.01A | GS_group |
| TCGA.FV.A2QQ.01A | GS_group |
| TCGA.DD.A113.01A | GS_group |
| TCGA.ED.A8O5.01A | GS_group |
| TCGA.CC.5258.01A | GS_group |
| TCGA.2Y.A9H3.01A | GS_group |
| TCGA.RC.A7SK.01A | GS_group |
| TCGA.ED.A459.01A | GS_group |
| TCGA.2Y.A9GU.01A | GS_group |
| TCGA.ZP.A9D1.01A | GS_group |
| TCGA.CC.A7IJ.01A | GS_group |
| TCGA.BC.4073.01B | GS_group |
| TCGA.DD.AACK.01A | GS_group |
| TCGA.5C.A9VG.01A | GS_group |
| TCGA.CC.A8HV.01A | GS_group |
| TCGA.CC.A7IG.01A | GS_group |
| TCGA.CC.A7II.01A | GS_group |
| TCGA.CC.A7IE.01A | GS_group |
| TCGA.DD.A3A9.01A | GS_group |
| TCGA.WQ.A9G7.01A | GS_group |
| TCGA.4R.AA8I.01A | GS_group |
| TCGA.XR.A8TE.01A | GU_group |
| TCGA.DD.A4NP.01A | GU_group |
| TCGA.G3.A5SI.01A | GU_group |
| TCGA.DD.A39W.01A | GU_group |
| TCGA.KR.A7K8.01A | GU_group |
| TCGA.EP.A12J.01A | GU_group |
| TCGA.DD.AACS.01A | GU_group |
| TCGA.DD.AACO.01A | GU_group |
| TCGA.LG.A9QC.01A | GU_group |
| TCGA.DD.AAE1.01A | GU_group |
| TCGA.XR.A8TD.01A | GU_group |
| TCGA.DD.A3A3.01A | GU_group |
| TCGA.G3.AAV2.01A | GU_group |
| TCGA.KR.A7K2.01A | GU_group |
| TCGA.G3.AAV1.01A | GU_group |
| TCGA.DD.A1EJ.01A | GU_group |
| TCGA.KR.A7K7.01A | GU_group |
| TCGA.WQ.AB4B.01A | GU_group |
| TCGA.DD.AAVU.01A | GU_group |
| TCGA.DD.A3A5.01A | GU_group |
| TCGA.DD.AADJ.01A | GU_group |
| TCGA.BC.A10Y.01A | GU_group |
| TCGA.UB.A7MC.01A | GU_group |
| TCGA.DD.AAE4.01A | GU_group |
| TCGA.DD.A3A2.01A | GU_group |
| TCGA.DD.A73D.01A | GU_group |
| TCGA.G3.A7M7.01A | GU_group |
| TCGA.DD.A39V.01A | GU_group |
| TCGA.G3.AAV6.01A | GU_group |
| TCGA.ES.A2HS.01A | GU_group |
| TCGA.DD.A39Z.01A | GU_group |
| TCGA.ZP.A9D4.01A | GU_group |
| TCGA.BC.A5W4.01A | GU_group |
| TCGA.5C.AAPD.01A | GU_group |
| TCGA.2Y.A9H4.01A | GU_group |
| TCGA.DD.AACY.01A | GU_group |
| TCGA.2Y.A9GY.01A | GU_group |
| TCGA.ZS.A9CG.01A | GU_group |
| TCGA.G3.A25U.01A | GU_group |
| TCGA.DD.A1EA.01A | GU_group |
| TCGA.DD.A4NK.01A | GU_group |
| TCGA.DD.A73B.01A | GU_group |
| TCGA.DD.A4NQ.01A | GU_group |
| TCGA.RC.A7SF.01A | GU_group |
| TCGA.DD.AAED.01A | GU_group |
| TCGA.G3.AAUZ.01A | GU_group |
| TCGA.DD.AAEK.01A | GU_group |
| TCGA.CC.A123.01A | GU_group |
| TCGA.RG.A7D4.01A | GU_group |
| TCGA.FV.A4ZQ.01A | GU_group |
| TCGA.DD.AAVP.01A | GU_group |
| TCGA.DD.AACE.01A | GU_group |
| TCGA.K7.A6G5.01A | GU_group |
| TCGA.DD.AAVX.01A | GU_group |
| TCGA.G3.A5SK.01A | GU_group |
| TCGA.G3.AAV3.01A | GU_group |
| TCGA.GJ.A6C0.01A | GU_group |
| TCGA.FV.A3R2.01A | GU_group |
| TCGA.DD.AADC.01A | GU_group |
| TCGA.2Y.A9H0.01A | GU_group |
| TCGA.G3.AAV5.01A | GU_group |
| TCGA.FV.A495.01A | GU_group |
| TCGA.BW.A5NO.01A | GU_group |
| TCGA.DD.AAEE.01A | GU_group |
| TCGA.DD.AADD.01A | GU_group |
| TCGA.DD.AACD.01A | GU_group |
| TCGA.DD.A1EL.01A | GU_group |
| TCGA.CC.A8HU.01A | GU_group |
| TCGA.EP.A26S.01A | GU_group |
| TCGA.5R.AA1C.01A | GU_group |
| TCGA.DD.A116.01A | GU_group |
| TCGA.DD.AAE6.01A | GU_group |
| TCGA.DD.AAW3.01A | GU_group |
| TCGA.DD.AAVY.01A | GU_group |
| TCGA.BC.A3KF.01A | GU_group |
| TCGA.FV.A496.01A | GU_group |
| TCGA.KR.A7K0.01A | GU_group |
| TCGA.DD.AADR.01A | GU_group |
| TCGA.ZP.A9CV.01A | GU_group |
| TCGA.G3.A3CG.01A | GU_group |
| TCGA.DD.AADU.01A | GU_group |
| TCGA.FV.A4ZP.01A | GU_group |
| TCGA.DD.AAD0.01A | GU_group |
| TCGA.DD.AACA.01A | GU_group |
| TCGA.DD.AACG.01A | GU_group |
| TCGA.DD.AADN.01A | GU_group |
| TCGA.DD.AACV.01A | GU_group |
| TCGA.DD.AACB.01A | GU_group |
| TCGA.BC.A217.01A | GU_group |
| TCGA.DD.AAE9.01A | GU_group |
| TCGA.DD.AAD6.01A | GU_group |
| TCGA.NI.A4U2.01A | GU_group |
| TCGA.DD.AACJ.01A | GU_group |
| TCGA.CC.A7IL.01A | GU_group |
| TCGA.G3.A5SL.01A | GU_group |
| TCGA.DD.A4NF.01A | GU_group |
| TCGA.DD.AAEI.01A | GU_group |
| TCGA.LG.A9QD.01A | GU_group |
| TCGA.DD.AAW1.01A | GU_group |
| TCGA.MI.A75C.01A | GU_group |
| TCGA.ZS.A9CE.01A | GU_group |
| TCGA.DD.A3A8.01A | GU_group |
| TCGA.G3.AAV4.01A | GU_group |
| TCGA.DD.AACX.01A | GU_group |
| TCGA.DD.AAD8.01A | GU_group |
| TCGA.DD.A3A7.01A | GU_group |
| TCGA.G3.A25S.01A | GU_group |
| TCGA.CC.A7IF.01A | GU_group |
| TCGA.DD.AADQ.01A | GU_group |
| TCGA.DD.A73E.01A | GU_group |
| TCGA.BC.A10U.01A | GU_group |
| TCGA.DD.AACF.01A | GU_group |
| TCGA.DD.A11D.01A | GU_group |
| TCGA.G3.A6UC.01A | GU_group |
| TCGA.DD.AAEB.01A | GU_group |
| TCGA.MI.A75H.01A | GU_group |
| TCGA.BC.A69H.01A | GU_group |
| TCGA.DD.AAD5.01A | GU_group |
| TCGA.CC.A5UE.01A | GU_group |
| TCGA.2Y.A9H1.01A | GU_group |
| TCGA.3K.AAZ8.01A | GU_group |
| TCGA.DD.AAW2.01A | GU_group |
| TCGA.CC.5259.01A | GU_group |
| TCGA.WJ.A86L.01A | GU_group |
| TCGA.DD.A1EB.01A | GU_group |
| TCGA.2Y.A9HA.01A | GU_group |
| TCGA.DD.AADS.01A | GU_group |
| TCGA.DD.AADG.01A | GU_group |
| TCGA.XR.A8TF.01A | GU_group |
| TCGA.DD.AAE3.01A | GU_group |
| TCGA.CC.A9FW.01A | GU_group |
| TCGA.DD.AACZ.01A | GU_group |
| TCGA.DD.AACP.01A | GU_group |
| TCGA.DD.AADM.01A | GU_group |
| TCGA.MI.A75I.01A | GU_group |
| TCGA.G3.AAV0.01A | GU_group |
| TCGA.DD.AAEA.01A | GU_group |
| TCGA.RC.A6M4.01A | GU_group |
| TCGA.BC.A10Z.01A | GU_group |
| TCGA.RC.A6M6.01A | GU_group |
| TCGA.G3.A7M5.01A | GU_group |
| TCGA.LG.A6GG.01A | GU_group |
| TCGA.CC.A8HT.01A | GU_group |
| TCGA.DD.AACT.01A | GU_group |
| TCGA.G3.A3CK.01A | GU_group |
| TCGA.DD.AADO.01A | GU_group |
| TCGA.CC.A5UD.01A | GU_group |
| TCGA.DD.AACQ.01A | GU_group |
| TCGA.DD.AADF.01A | GU_group |
| TCGA.MI.A75G.01A | GU_group |
| TCGA.DD.AAE7.01A | GU_group |
| TCGA.DD.A1EE.01A | GU_group |
| TCGA.ED.A7PZ.01A | GU_group |
| TCGA.CC.A7IK.01A | GU_group |
| TCGA.DD.AACL.01A | GU_group |
| TCGA.DD.AACI.01A | GU_group |
| TCGA.DD.AAC8.01A | GU_group |
| TCGA.CC.A7IH.01A | GU_group |
| TCGA.UB.A7MB.01A | GU_group |
