# Supplementary material for: A Genomic Instability-Related Long Noncoding RNA Signature for Predicting Hepatocellular Carcinoma Prognosis
Source: J Oncol. 2022 Aug 29;2022:3090523. doi: 10.1155/2022/3090523 (PMC9444385; doi:10.1155/2022/3090523)
Supplement: Supplementary Materials — Table S1: The overall clinical characteristics of 353 patients Table S2: The information of HM-group and LM-group Table S3: The information of 52 up-regulated lncRNAs and 83 down-regulated lncRNAs Table S4: The information of GS-group and GU-group Table S5: The information of high-risk group and low-risk group Table S6: The concrete clinical information of 353 patients. [file 3090523.f1.zip › 3090523.f1/TableS6.docx]

| sample | OS | OS.time | group | Age | Gender | stage | grade | T | N | M | RiskScore |
| --- | --- | --- | --- | --- | --- | --- | --- | --- | --- | --- | --- |
| TCGA.2Y.A9H5.01A | 1 | 555 | LOW | 21818 | female | stage i | G3 | T1 | N0 | NA | 0.96782033 |
| TCGA.2Y.A9GW.01A | 1 | 1271 | LOW | 23587 | male | stage i | G2 | T1 | N0 | NA | 1.02118071 |
| TCGA.DD.AA3A.01A | 1 | 410 | LOW | 29712 | female | stage i | G4 | T1 | N0 | NA | 1.07044647 |
| TCGA.2Y.A9H9.01A | 0 | 697 | LOW | 25670 | male | stage i | G2 | T1 | N0 | NA | 1.18722037 |
| TCGA.2Y.A9H7.01A | 0 | 1168 | LOW | 29752 | female | stage i | G2 | T1 | N0 | NA | 1.2165797 |
| TCGA.FV.A2QQ.01A | 0 | 729 | LOW | 29354 | male | stage i | G2 | T1 | N0 | NA | 1.23341504 |
| TCGA.K7.A6G5.01A | 0 | 512 | LOW | 24233 | male | stage i | G2 | T1 | N0 | NA | 1.35746113 |
| TCGA.2Y.A9H2.01A | 0 | 1731 | LOW | 23653 | female | stage i | G3 | T1 | N0 | NA | 1.38224267 |
| TCGA.2Y.A9H4.01A | 0 | 1452 | LOW | 25096 | male | stage i | G2 | T1 | N0 | NA | 1.58786283 |
| TCGA.EP.A26S.01A | 0 | 608 | HIGH | 25677 | male | stage i | G2 | T1 | N0 | NA | 1.6547787 |
| TCGA.GJ.A9DB.01A | 1 | 67 | HIGH | 25020 | male | stage i | G2 | T1 | N0 | NA | 1.79939631 |
| TCGA.MR.A8JO.01A | 0 | 330 | HIGH | 12669 | male | stage i | G3 | T1 | N0 | NA | 1.91435314 |
| TCGA.BC.A10Z.01A | 1 | 34 | HIGH | 22913 | female | stage i | G2 | T1 | N0 | NA | 2.8460884 |
| TCGA.UB.A7MD.01A | 1 | 52 | HIGH | 24717 | male | stage i | G3 | T1 | N0 | NA | 2.96885104 |
| TCGA.FV.A3I1.01A | 1 | 247 | HIGH | NA | female | stage ii | G2 | T2 | N0 | NA | 2.13430888 |
| TCGA.XR.A8TE.01A | 0 | 925 | HIGH | 5862 | male | stage iii | G1 | T3 | N0 | NA | 1.94287275 |
| TCGA.BC.4073.01B | 0 | 849 | HIGH | 26795 | male | stage iii | G3 | T3 | N0 | NA | 2.55312649 |
| TCGA.BC.A10X.01A | 1 | 770 | LOW | 19140 | female | stage iii | G2 | T3 | N0 | NA | 0.36493969 |
| TCGA.UB.A7MC.01A | 0 | 500 | HIGH | 21733 | male | stage iii | G3 | T3 | N0 | NA | 2.21989223 |
| TCGA.YA.A8S7.01A | 1 | 412 | HIGH | 25188 | male | stage iii | G3 | T3 | N0 | NA | 2.59982646 |
| TCGA.DD.A119.01A | 1 | 223 | HIGH | 14613 | male | stage iv | G3 | T3 | N0 | M1 | 2.32102126 |
| TCGA.DD.A3A9.01A | 1 | 931 | LOW | 23526 | female | stage iv | G2 | T4 | N0 | M1 | 0.99520411 |
| TCGA.DD.A1EK.01A | 1 | 558 | HIGH | 23437 | female | stage iv | G2 | T4 | N0 | M1 | 1.92521759 |
| TCGA.DD.AAE1.01A | 0 | 552 | LOW | 19073 | male | stage i | G3 | T1 | N0 | M0 | -0.1531606 |
| TCGA.DD.AAD1.01A | 0 | 564 | LOW | 18794 | female | stage i | G4 | T1 | N0 | M0 | 0.0089761 |
| TCGA.DD.AAVW.01A | 0 | 2317 | LOW | 12986 | male | stage i | G2 | T1 | N0 | M0 | 0.03802014 |
| TCGA.DD.AADJ.01A | 0 | 1066 | LOW | 25863 | female | stage i | G3 | T1 | N0 | M0 | 0.13963246 |
| TCGA.DD.AAW0.01A | 0 | 2015 | LOW | 19981 | male | stage i | G2 | T1 | N0 | M0 | 0.22857372 |
| TCGA.DD.AAD2.01A | 0 | 658 | LOW | 24221 | male | stage i | G2 | T1 | N0 | M0 | 0.25738656 |
| TCGA.G3.A3CI.01A | 0 | 180 | LOW | 26224 | male | stage i | G2 | T1 | N0 | M0 | 0.27119575 |
| TCGA.DD.AACW.01A | 0 | 1424 | LOW | 15872 | male | stage i | G3 | T1 | N0 | M0 | 0.29942539 |
| TCGA.DD.AADI.01A | 0 | 1085 | LOW | 15928 | female | stage i | G3 | T1 | N0 | M0 | 0.31493273 |
| TCGA.DD.A11B.01A | 1 | 14 | LOW | 26857 | male | stage i | G2 | T1 | N0 | M0 | 0.43195033 |
| TCGA.DD.AAEA.01A | 0 | 575 | LOW | 24027 | male | stage i | G3 | T1 | N0 | M0 | 0.43277513 |
| TCGA.DD.AACT.01A | 0 | 1562 | LOW | 25292 | female | stage i | G2 | T1 | N0 | M0 | 0.44569164 |
| TCGA.DD.AADA.01A | 0 | 1233 | LOW | 24288 | female | stage i | G3 | T1 | N0 | M0 | 0.47661513 |
| TCGA.DD.AAVQ.01A | 0 | 2728 | LOW | 13911 | male | stage i | G2 | T1 | N0 | M0 | 0.55229811 |
| TCGA.DD.AACE.01A | 0 | 2184 | LOW | 22727 | male | stage i | G3 | T1 | N0 | M0 | 0.55439745 |
| TCGA.DD.AACN.01A | 0 | 1302 | LOW | 12007 | male | stage i | G3 | T1 | N0 | M0 | 0.57320939 |
| TCGA.DD.AAVS.01A | 0 | 1823 | LOW | 20586 | male | stage i | G2 | T1 | N0 | M0 | 0.59389844 |
| TCGA.DD.AACO.01A | 0 | 1876 | LOW | 14796 | male | stage i | G3 | T1 | N0 | M0 | 0.60050917 |
| TCGA.RC.A7SF.01A | 0 | 579 | LOW | 24432 | male | stage i | G2 | T1 | N0 | M0 | 0.6143039 |
| TCGA.DD.AAEB.01A | 0 | 478 | LOW | 22121 | male | stage i | G2 | T1 | N0 | M0 | 0.62826695 |
| TCGA.DD.AAEH.01A | 0 | 784 | LOW | 26894 | male | stage i | G2 | T1 | N0 | M0 | 0.64180967 |
| TCGA.DD.A73F.01A | 0 | 1085 | LOW | 28234 | female | stage i | G1 | T1 | N0 | M0 | 0.66720215 |
| TCGA.DD.A4NL.01A | 0 | 1711 | LOW | 16874 | male | stage i | G1 | T1 | N0 | M0 | 0.67490358 |
| TCGA.KR.A7K2.01A | 0 | 829 | LOW | 23699 | male | stage i | G1 | T1 | N0 | M0 | 0.73325293 |
| TCGA.DD.AAE4.01A | 0 | 608 | LOW | 18009 | female | stage i | G1 | T1 | N0 | M0 | 0.73928366 |
| TCGA.DD.AACC.01A | 1 | 1685 | LOW | 22499 | male | stage i | G2 | T1 | N0 | M0 | 0.77404002 |
| TCGA.DD.A4NS.01A | 1 | 2456 | LOW | 22638 | female | stage i | G2 | T1 | N0 | M0 | 0.77689583 |
| TCGA.DD.AAED.01A | 0 | 763 | LOW | 18722 | male | stage i | G3 | T1 | N0 | M0 | 0.79099899 |
| TCGA.DD.AAE7.01A | 0 | 644 | LOW | 26568 | male | stage i | G2 | T1 | N0 | M0 | 0.79556946 |
| TCGA.DD.AAC9.01A | 0 | 347 | LOW | 18911 | male | stage i | G2 | T1 | N0 | M0 | 0.79901118 |
| TCGA.DD.AAEI.01A | 0 | 1531 | LOW | 26465 | male | stage i | G2 | T1 | N0 | M0 | 0.82142681 |
| TCGA.DD.A73A.01A | 0 | 728 | LOW | 26136 | male | stage i | G2 | T1 | N0 | M0 | 0.83824725 |
| TCGA.DD.AAVR.01A | 0 | 2513 | LOW | 16157 | male | stage i | G2 | T1 | N0 | M0 | 0.86198911 |
| TCGA.DD.AAE6.01A | 0 | 141 | LOW | 21764 | female | stage i | G2 | T1 | N0 | M0 | 0.90085168 |
| TCGA.DD.AAD3.01A | 0 | 1295 | LOW | 15706 | male | stage i | G2 | T1 | N0 | M0 | 0.93768485 |
| TCGA.DD.A73G.01A | 0 | 3478 | LOW | 26949 | female | stage i | G3 | T1 | N0 | M0 | 0.94358453 |
| TCGA.DD.A4ND.01A | 0 | 2746 | LOW | 20782 | female | stage i | G3 | T1 | N0 | M0 | 0.96791939 |
| TCGA.5C.A9VH.01A | 0 | 322 | LOW | 25608 | male | stage i | G2 | T1 | N0 | M0 | 0.98274182 |
| TCGA.DD.A1EF.01A | 1 | 394 | LOW | 21165 | female | stage i | G3 | T1 | N0 | M0 | 1.01629081 |
| TCGA.DD.AADW.01A | 0 | 587 | LOW | 17781 | male | stage i | G3 | T1 | N0 | M0 | 1.04905749 |
| TCGA.DD.A11C.01A | 0 | 662 | LOW | 25403 | male | stage i | G3 | T1 | N0 | M0 | 1.06817642 |
| TCGA.DD.AADS.01A | 0 | 474 | LOW | 23227 | male | stage i | G2 | T1 | N0 | M0 | 1.07381898 |
| TCGA.DD.A3A3.01A | 1 | 535 | LOW | 16708 | male | stage i | G2 | T1 | N0 | M0 | 1.10165633 |
| TCGA.DD.A4NP.01A | 0 | 3308 | LOW | 11838 | male | stage i | G3 | T1 | N0 | M0 | 1.13680981 |
| TCGA.G3.A25V.01A | 0 | 860 | LOW | 25144 | male | stage i | G2 | T1 | N0 | M0 | 1.1410678 |
| TCGA.DD.AACB.01A | 0 | 2324 | LOW | 27103 | female | stage i | G3 | T1 | N0 | M0 | 1.14544667 |
| TCGA.DD.A1ED.01A | 0 | 2301 | LOW | 25039 | male | stage i | G1 | T1 | N0 | M0 | 1.16815817 |
| TCGA.DD.A3A2.01A | 1 | 2131 | LOW | 27930 | female | stage i | G1 | T1 | N0 | M0 | 1.17318266 |
| TCGA.DD.AAD0.01A | 0 | 137 | LOW | 26781 | female | stage i | G2 | T1 | N0 | M0 | 1.17464681 |
| TCGA.DD.A73B.01A | 1 | 283 | LOW | 26390 | female | stage i | G2 | T1 | N0 | M0 | 1.17803774 |
| TCGA.G3.AAV0.01A | 0 | 476 | LOW | 21249 | male | stage i | G2 | T1 | N0 | M0 | 1.23394589 |
| TCGA.DD.AAW2.01A | 0 | 1855 | LOW | 25420 | male | stage i | G2 | T1 | N0 | M0 | 1.25926986 |
| TCGA.G3.A25Y.01A | 1 | 452 | LOW | 19055 | female | stage i | G3 | T1 | N0 | M0 | 1.26341014 |
| TCGA.DD.AAE3.01A | 0 | 566 | LOW | 18271 | male | stage i | G2 | T1 | N0 | M0 | 1.26519511 |
| TCGA.DD.A1EB.01A | 0 | 2017 | LOW | 26471 | female | stage i | G2 | T1 | N0 | M0 | 1.26719495 |
| TCGA.DD.A4NN.01A | 1 | 899 | LOW | 20548 | female | stage i | G3 | T1 | N0 | M0 | 1.26855598 |
| TCGA.DD.AACF.01A | 1 | 365 | LOW | 25172 | male | stage i | G3 | T1 | N0 | M0 | 1.28620422 |
| TCGA.DD.A1EI.01A | 0 | 183 | LOW | 17097 | male | stage i | G2 | T1 | N0 | M0 | 1.31058809 |
| TCGA.DD.AAE9.01A | 0 | 722 | LOW | 25451 | male | stage i | G3 | T1 | N0 | M0 | 1.40121477 |
| TCGA.G3.AAUZ.01A | 0 | 480 | LOW | 17760 | male | stage i | G2 | T1 | N0 | M0 | 1.41106074 |
| TCGA.DD.AACY.01A | 0 | 1450 | LOW | 22332 | male | stage i | G3 | T1 | N0 | M0 | 1.41361661 |
| TCGA.G3.A25Z.01A | 0 | 655 | LOW | 21509 | male | stage i | G2 | T1 | N0 | M0 | 1.41852028 |
| TCGA.DD.A11D.01A | 1 | 1560 | LOW | 21003 | female | stage i | G2 | T1 | N0 | M0 | 1.42122395 |
| TCGA.DD.AACA.01A | 0 | 2301 | LOW | 23973 | male | stage i | G3 | T1 | N0 | M0 | 1.45550945 |
| TCGA.DD.AACU.01A | 0 | 1567 | LOW | 21606 | male | stage i | G3 | T1 | N0 | M0 | 1.46412161 |
| TCGA.BD.A3EP.01A | 0 | 409 | LOW | 27756 | female | stage i | G2 | T1 | N0 | M0 | 1.53565744 |
| TCGA.DD.AAE2.01A | 0 | 638 | LOW | 18695 | male | stage i | G3 | T1 | N0 | M0 | 1.54139603 |
| TCGA.DD.A1EC.01A | 0 | 602 | LOW | 7363 | female | stage i | G3 | T1 | N0 | M0 | 1.54550823 |
| TCGA.DD.AAVZ.01A | 0 | 1900 | LOW | 14005 | male | stage i | G2 | T1 | N0 | M0 | 1.55044654 |
| TCGA.DD.AAVP.01A | 0 | 2752 | LOW | 17833 | male | stage i | G1 | T1 | N0 | M0 | 1.57924182 |
| TCGA.DD.A4NB.01A | 0 | 989 | HIGH | 9426 | male | stage i | G2 | T1 | N0 | M0 | 1.60737233 |
| TCGA.DD.AADP.01A | 0 | 458 | HIGH | 16760 | male | stage i | G3 | T1 | N0 | M0 | 1.66377825 |
| TCGA.DD.AAW3.01A | 0 | 1633 | HIGH | 25254 | male | stage i | G2 | T1 | N0 | M0 | 1.68887178 |
| TCGA.MI.A75C.01A | 0 | 291 | HIGH | 23441 | male | stage i | G3 | T1 | N0 | M0 | 1.7407857 |
| TCGA.DD.A4NO.01A | 0 | 2245 | HIGH | 24090 | male | stage i | G1 | T1 | N0 | M0 | 1.74631284 |
| TCGA.DD.AACS.01A | 0 | 1804 | HIGH | 14473 | male | stage i | G3 | T1 | N0 | M0 | 1.75082531 |
| TCGA.DD.AADO.01A | 0 | 453 | HIGH | 20139 | male | stage i | G3 | T1 | N0 | M0 | 1.76612087 |
| TCGA.DD.AADR.01A | 0 | 2028 | HIGH | 21217 | male | stage i | G3 | T1 | N0 | M0 | 1.77328688 |
| TCGA.G3.A3CG.01A | 0 | 673 | HIGH | 29432 | male | stage i | G2 | T1 | N0 | M0 | 1.79967058 |
| TCGA.G3.A25U.01A | 0 | 1636 | HIGH | 23207 | female | stage i | G3 | T1 | N0 | M0 | 1.80367927 |
| TCGA.DD.AACV.01A | 0 | 1531 | HIGH | 19660 | male | stage i | G3 | T1 | N0 | M0 | 1.81146391 |
| TCGA.KR.A7K8.01A | 0 | 906 | HIGH | 20914 | male | stage i | G1 | T1 | N0 | M0 | 1.81250487 |
| TCGA.DD.AADL.01A | 0 | 636 | HIGH | 21512 | male | stage i | G4 | T1 | N0 | M0 | 1.83912133 |
| TCGA.G3.A3CK.01A | 0 | 585 | HIGH | 22462 | male | stage i | G2 | T1 | N0 | M0 | 1.89124692 |
| TCGA.DD.AADB.01A | 0 | 1242 | HIGH | 18728 | male | stage i | G4 | T1 | N0 | M0 | 1.96734038 |
| TCGA.DD.AADV.01A | 0 | 574 | HIGH | 18300 | male | stage i | G3 | T1 | N0 | M0 | 1.97931572 |
| TCGA.DD.AADF.01A | 1 | 115 | HIGH | 23614 | female | stage i | G4 | T1 | N0 | M0 | 1.99827574 |
| TCGA.DD.AADD.01A | 0 | 1231 | HIGH | 18752 | male | stage i | G4 | T1 | N0 | M0 | 2.00155966 |
| TCGA.DD.A73E.01A | 0 | 44 | HIGH | 24377 | male | stage i | G1 | T1 | N0 | M0 | 2.00330096 |
| TCGA.DD.AACP.01A | 0 | 415 | HIGH | 23728 | male | stage i | G3 | T1 | N0 | M0 | 2.01971653 |
| TCGA.FV.A2QR.01A | 1 | 581 | HIGH | NA | male | stage i | G1 | T1 | N0 | M0 | 2.02285072 |
| TCGA.G3.AAV2.01A | 0 | 372 | HIGH | 18363 | male | stage i | G1 | T1 | N0 | M0 | 2.05650651 |
| TCGA.DD.AAD8.01A | 0 | 1219 | HIGH | 26781 | female | stage i | G2 | T1 | N0 | M0 | 2.11157879 |
| TCGA.RC.A7SK.01A | 0 | 472 | HIGH | 21814 | male | stage i | G3 | T1 | N0 | M0 | 2.22017951 |
| TCGA.DD.AAEG.01A | 0 | 719 | HIGH | 21853 | female | stage i | G3 | T1 | N0 | M0 | 2.24746159 |
| TCGA.DD.A4NR.01A | 1 | 9 | HIGH | 31386 | female | stage i | G3 | T1 | N0 | M0 | 2.30547019 |
| TCGA.DD.A4NF.01A | 0 | 942 | HIGH | 26330 | male | stage i | G2 | T1 | N0 | M0 | 2.32067473 |
| TCGA.DD.AADY.01A | 0 | 555 | HIGH | 20233 | female | stage i | G2 | T1 | N0 | M0 | 2.33825333 |
| TCGA.DD.AACK.01A | 0 | 9 | HIGH | 25796 | male | stage i | G2 | T1 | N0 | M0 | 2.35516601 |
| TCGA.KR.A7K0.01A | 1 | 65 | HIGH | 23974 | male | stage i | G1 | T1 | N0 | M0 | 2.35545388 |
| TCGA.DD.AACL.01A | 1 | 107 | HIGH | 24355 | female | stage i | G3 | T1 | N0 | M0 | 2.41056417 |
| TCGA.G3.AAV4.01A | 1 | 27 | HIGH | 30466 | female | stage i | G1 | T1 | N0 | M0 | 2.41832438 |
| TCGA.RC.A7S9.01A | 0 | 640 | HIGH | 17403 | female | stage i | G3 | T1 | N0 | M0 | 2.46353281 |
| TCGA.G3.A25S.01A | 1 | 416 | HIGH | 23571 | male | stage i | G2 | T1 | N0 | M0 | 2.51383047 |
| TCGA.DD.AADC.01A | 1 | 425 | HIGH | 19527 | male | stage i | G3 | T1 | N0 | M0 | 2.57314186 |
| TCGA.DD.AACD.01A | 1 | 381 | HIGH | 17828 | male | stage i | G4 | T1 | N0 | M0 | 2.65025156 |
| TCGA.DD.AAEE.01A | 0 | 810 | HIGH | 20396 | male | stage i | G4 | T1 | N0 | M0 | 2.67999735 |
| TCGA.DD.AAD5.01A | 0 | 1345 | HIGH | 19748 | male | stage i | G3 | T1 | N0 | M0 | 2.85643369 |
| TCGA.DD.A11A.01A | 1 | 79 | HIGH | 24646 | male | stage i | G3 | T1 | N0 | M0 | 3.21019592 |
| TCGA.DD.AAC8.01A | 1 | 16 | HIGH | 26322 | male | stage i | G3 | T1 | N0 | M0 | 3.21950781 |
| TCGA.DD.AACZ.01A | 1 | 171 | HIGH | 23230 | female | stage i | G4 | T1 | N0 | M0 | 3.61239663 |
| TCGA.RC.A7SH.01A | 0 | 468 | LOW | 15560 | male | stage ii | G3 | T2 | N0 | M0 | 0.47470832 |
| TCGA.DD.AAEK.01A | 0 | 1067 | LOW | 18757 | male | stage ii | G3 | T2 | N0 | M0 | 0.55659297 |
| TCGA.ED.A5KG.01A | 0 | 854 | LOW | 22258 | female | stage ii | G2 | T2 | N0 | M0 | 0.68066924 |
| TCGA.ED.A4XI.01A | 0 | 819 | LOW | 21497 | male | stage ii | G3 | T2 | N0 | M0 | 0.70515832 |
| TCGA.DD.AACI.01A | 0 | 1618 | LOW | 25236 | male | stage ii | G3 | T2 | N0 | M0 | 0.7935272 |
| TCGA.G3.A5SI.01A | 1 | 768 | LOW | 16126 | male | stage ii | G2 | T2 | N0 | M0 | 1.04060896 |
| TCGA.DD.A1EA.01A | 0 | 2415 | LOW | 24925 | male | stage ii | G2 | T2 | N0 | M0 | 1.04592675 |
| TCGA.DD.AADK.01A | 0 | 1049 | LOW | 25046 | female | stage ii | G3 | T2 | N0 | M0 | 1.04593481 |
| TCGA.CC.A9FS.01A | 0 | 211 | LOW | 20157 | male | stage ii | G2 | T2 | N0 | M0 | 1.11667506 |
| TCGA.DD.AADM.01A | 1 | 12 | LOW | 21349 | male | stage ii | G3 | T2 | N0 | M0 | 1.2834704 |
| TCGA.DD.AADQ.01A | 0 | 436 | LOW | 21756 | male | stage ii | G3 | T2 | N0 | M0 | 1.28901546 |
| TCGA.CC.A7IJ.01A | 0 | 382 | LOW | 20751 | male | stage ii | G3 | T2 | N0 | M0 | 1.28929655 |
| TCGA.DD.AAVV.01A | 0 | 2455 | LOW | 20751 | male | stage ii | G3 | T2 | N0 | M0 | 1.34750027 |
| TCGA.G3.AAV3.01A | 0 | 412 | LOW | 21449 | female | stage ii | G2 | T2 | N0 | M0 | 1.36589072 |
| TCGA.DD.A4NJ.01A | 0 | 928 | LOW | 19964 | female | stage ii | G2 | T2 | N0 | M0 | 1.4066899 |
| TCGA.DD.AAVX.01A | 0 | 1718 | LOW | 14016 | male | stage ii | G2 | T2 | N0 | M0 | 1.4391309 |
| TCGA.FV.A23B.01A | 1 | 1852 | LOW | 25788 | female | stage ii | NA | T2 | N0 | M0 | 1.47357261 |
| TCGA.5R.AAAM.01A | 1 | 46 | LOW | 24020 | female | stage ii | G2 | T2 | N0 | M0 | 1.48706134 |
| TCGA.G3.AAV5.01A | 0 | 354 | LOW | 24655 | male | stage ii | G2 | T2 | N0 | M0 | 1.48982468 |
| TCGA.RC.A7SB.01A | 0 | 588 | LOW | 19698 | male | stage ii | G2 | T2 | N0 | M0 | 1.49170255 |
| TCGA.RG.A7D4.01A | 0 | 1098 | LOW | 25302 | male | stage ii | G2 | T2 | N0 | M0 | 1.50374928 |
| TCGA.5C.A9VG.01A | 0 | 328 | LOW | 21273 | male | stage ii | G2 | T2 | N0 | M0 | 1.52218232 |
| TCGA.DD.AACX.01A | 0 | 170 | LOW | 24329 | male | stage ii | G3 | T2 | N0 | M0 | 1.58986903 |
| TCGA.5C.AAPD.01A | 0 | 20 | HIGH | 22552 | male | stage ii | G1 | T2 | N0 | M0 | 1.59956589 |
| TCGA.DD.A3A8.01A | 1 | 11 | HIGH | 27524 | male | stage ii | G2 | T2 | N0 | M0 | 1.59972488 |
| TCGA.5R.AA1C.01A | 0 | 520 | HIGH | 21073 | male | stage ii | G2 | T2 | N0 | M0 | 1.69895164 |
| TCGA.DD.A118.01A | 0 | 3437 | HIGH | 28406 | female | stage ii | G2 | T2 | N0 | M0 | 1.70209738 |
| TCGA.DD.AACG.01A | 1 | 469 | HIGH | 19174 | male | stage ii | G4 | T2 | N0 | M0 | 1.83645635 |
| TCGA.MI.A75G.01A | 0 | 698 | HIGH | 23284 | male | stage ii | G2 | T2 | N0 | M0 | 1.86543811 |
| TCGA.DD.AADU.01A | 0 | 554 | HIGH | 21940 | male | stage ii | G3 | T2 | N0 | M0 | 1.87665273 |
| TCGA.DD.A113.01A | 0 | 2425 | HIGH | 20188 | female | stage ii | G3 | T2 | N0 | M0 | 1.88936427 |
| TCGA.DD.AAVU.01A | 0 | 2202 | HIGH | 16889 | male | stage ii | G2 | T2 | N0 | M0 | 1.91351062 |
| TCGA.DD.A3A6.01A | 1 | 3258 | HIGH | 26319 | female | stage ii | G2 | T2 | N0 | M0 | 2.00221194 |
| TCGA.ED.A7XP.01A | 0 | 400 | HIGH | 19504 | female | stage ii | G3 | T2 | N0 | M0 | 2.02098647 |
| TCGA.DD.AACQ.01A | 1 | 432 | HIGH | 18429 | male | stage ii | G3 | T2 | N0 | M0 | 2.02551681 |
| TCGA.CC.5258.01A | 1 | 129 | HIGH | 17586 | male | stage ii | G2 | T2 | N0 | M0 | 2.08014819 |
| TCGA.G3.AAV7.01A | 0 | 361 | HIGH | 14229 | male | stage ii | G2 | T2 | N0 | M0 | 2.12528868 |
| TCGA.CC.A8HV.01A | 1 | 279 | HIGH | 18935 | female | stage ii | G2 | T2 | N0 | M0 | 2.27067815 |
| TCGA.ED.A459.01A | 0 | 910 | HIGH | 17402 | male | stage ii | G2 | T2 | N0 | M0 | 2.27335922 |
| TCGA.KR.A7K7.01A | 0 | 951 | HIGH | 22587 | female | stage ii | G1 | T2 | N0 | M0 | 2.62936347 |
| TCGA.CC.A7IG.01A | 1 | 299 | HIGH | 17453 | male | stage ii | G2 | T2 | N0 | M0 | 2.68928979 |
| TCGA.DD.A4NQ.01A | 1 | 373 | HIGH | 22111 | male | stage ii | G3 | T2 | N0 | M0 | 2.80315498 |
| TCGA.DD.AACJ.01A | 0 | 2102 | HIGH | 27528 | male | stage ii | G2 | T2 | N0 | M0 | 2.8771625 |
| TCGA.DD.A1EL.01A | 1 | 415 | HIGH | 8573 | male | stage ii | G3 | T2 | N0 | M0 | 3.69540611 |
| TCGA.DD.AACH.01A | 1 | 195 | HIGH | 25544 | male | stage ii | G3 | T2 | N0 | M0 | 4.00125544 |
| TCGA.UB.A7MA.01A | 0 | 848 | HIGH | 22843 | female | stage ii | G2 | T2 | N0 | M0 | 2.24788689 |
| TCGA.DD.A39W.01A | 1 | 827 | LOW | 10699 | female | stage iii | G2 | T3 | N0 | M0 | 1.22513519 |
| TCGA.DD.A3A5.01A | 1 | 3125 | HIGH | 24288 | female | stage iii | G2 | T3 | N0 | M0 | 1.63044127 |
| TCGA.DD.A1EH.01A | 0 | 1495 | HIGH | 8499 | male | stage iii | G3 | T3 | N0 | M0 | 2.02224419 |
| TCGA.DD.A4NV.01A | 0 | 2398 | LOW | 22438 | male | stage iii | G1 | T3 | N0 | M0 | 0.838505 |
| TCGA.DD.A4NK.01A | 1 | 1210 | LOW | 29244 | female | stage iii | G2 | T3 | N0 | M0 | 1.01001617 |
| TCGA.CC.A7IF.01A | 1 | 649 | LOW | 21845 | male | stage iii | G1 | T3 | N0 | M0 | 1.02143199 |
| TCGA.DD.A115.01A | 1 | 2542 | LOW | 19697 | male | stage iii | G2 | T3 | N0 | M0 | 1.25854563 |
| TCGA.DD.AAVY.01A | 0 | 1970 | LOW | 20709 | male | stage iii | G2 | T3 | N0 | M0 | 1.32019819 |
| TCGA.CC.A123.01A | 0 | 219 | LOW | 9107 | female | stage iii | G1 | T3 | N0 | M0 | 1.44256231 |
| TCGA.CC.A3M9.01A | 1 | 300 | HIGH | 16460 | male | stage iii | G3 | T3 | N0 | M0 | 1.5923538 |
| TCGA.DD.AAW1.01A | 0 | 1989 | HIGH | 20288 | male | stage iii | G2 | T3 | N0 | M0 | 1.65597478 |
| TCGA.CC.A5UC.01A | 1 | 347 | HIGH | 23156 | male | stage iii | G3 | T3 | N0 | M0 | 1.66117267 |
| TCGA.CC.A7IE.01A | 1 | 217 | HIGH | 20985 | male | stage iii | G2 | T3 | N0 | M0 | 1.78976869 |
| TCGA.2Y.A9H0.01A | 0 | 3675 | HIGH | 18109 | male | stage iii | G1 | T3 | N0 | M0 | 1.81526511 |
| TCGA.CC.A7IL.01A | 1 | 278 | HIGH | 22319 | male | stage iii | G1 | T3 | N0 | M0 | 1.85313841 |
| TCGA.CC.A3MC.01A | 0 | 363 | HIGH | 20067 | male | stage iii | G2 | T3 | N0 | M0 | 1.93469476 |
| TCGA.CC.A3MB.01A | 1 | 315 | HIGH | 13255 | male | stage iii | G1 | T3 | N0 | M0 | 2.02091803 |
| TCGA.CC.A3MA.01A | 1 | 303 | HIGH | 22375 | male | stage iii | G2 | T3 | N0 | M0 | 2.07041208 |
| TCGA.G3.A25T.01A | 0 | 1553 | HIGH | 16461 | female | stage iii | G2 | T3 | N0 | M0 | 2.11709173 |
| TCGA.CC.A7IH.01A | 0 | 365 | HIGH | 21381 | male | stage iii | G1 | T3 | N0 | M0 | 2.16682728 |
| TCGA.CC.A9FW.01A | 0 | 248 | HIGH | 24891 | male | stage iii | G2 | T3 | N0 | M0 | 2.16920832 |
| TCGA.DD.A116.01A | 1 | 1622 | HIGH | 24934 | male | stage iii | G3 | T3 | N0 | M0 | 2.3491453 |
| TCGA.CC.A7II.01A | 0 | 399 | HIGH | 20079 | male | stage iii | G3 | T3 | N0 | M0 | 2.37905854 |
| TCGA.CC.A5UD.01A | 1 | 304 | HIGH | 16702 | male | stage iii | G2 | T3 | N0 | M0 | 2.60770054 |
| TCGA.DD.A1EE.01A | 1 | 349 | HIGH | 26858 | male | stage iii | G3 | T3 | N0 | M0 | 2.64846841 |
| TCGA.CC.A8HU.01A | 1 | 344 | HIGH | 14367 | female | stage iii | G3 | T3 | N0 | M0 | 2.67516495 |
| TCGA.CC.5264.01A | 1 | 102 | HIGH | 25979 | male | stage iii | G2 | T3 | N0 | M0 | 2.80422089 |
| TCGA.CC.A8HT.01A | 1 | 140 | HIGH | 27334 | male | stage iii | G2 | T3 | N0 | M0 | 2.81972431 |
| TCGA.DD.A3A4.01A | 1 | 612 | HIGH | 13739 | male | stage iii | G3 | T3 | N0 | M0 | 2.84440581 |
| TCGA.CC.5263.01A | 1 | 129 | HIGH | 12863 | male | stage iii | G1 | T3 | N0 | M0 | 3.33500708 |
| TCGA.CC.A7IK.01A | 1 | 262 | HIGH | 21552 | male | stage iii | G3 | T3 | N0 | M0 | 3.9676267 |
| TCGA.CC.A1HT.01A | 1 | 101 | HIGH | 18553 | male | stage iii | G3 | T3 | N0 | M0 | 4.58266056 |
| TCGA.XR.A8TD.01A | 0 | 1030 | HIGH | 17908 | female | stage iii | G3 | T3 | N0 | M0 | 1.67095425 |
| TCGA.LG.A9QD.01A | 0 | 366 | LOW | 24969 | male | stage iii | G2 | T3 | N0 | M0 | 0.3883207 |
| TCGA.DD.A73C.01A | 0 | 701 | LOW | 23782 | female | stage iii | G1 | T3 | N0 | M0 | 0.50531413 |
| TCGA.DD.AAE0.01A | 0 | 555 | LOW | 16732 | female | stage iii | G4 | T3 | N0 | M0 | 0.69289766 |
| TCGA.G3.A3CH.01A | 0 | 780 | LOW | 19473 | male | stage iii | G2 | T3 | N0 | M0 | 0.82700178 |
| TCGA.ED.A8O5.01A | 0 | 406 | LOW | 21648 | female | stage iii | G3 | T3 | N0 | M0 | 0.86021171 |
| TCGA.ED.A7XO.01A | 0 | 427 | LOW | 10828 | male | stage iii | G2 | T3 | N0 | M0 | 1.00006332 |
| TCGA.DD.A4NE.01A | 1 | 660 | LOW | 27549 | female | stage iii | G3 | T3 | N0 | M0 | 1.25668312 |
| TCGA.ED.A82E.01A | 0 | 408 | LOW | 22234 | female | stage iii | G2 | T3 | N0 | M0 | 1.34127088 |
| TCGA.ED.A66X.01A | 0 | 406 | LOW | 12947 | male | stage iii | G3 | T3 | N0 | M0 | 1.47744068 |
| TCGA.DD.AADG.01A | 0 | 1145 | LOW | 25887 | male | stage iii | G3 | T3 | N0 | M0 | 1.51187811 |
| TCGA.5R.AA1D.01A | 0 | 449 | LOW | 6545 | female | stage iii | G3 | T3 | N0 | M0 | 1.53745053 |
| TCGA.ED.A66Y.01A | 1 | 296 | HIGH | 18797 | female | stage iii | G3 | T3 | N0 | M0 | 1.6807626 |
| TCGA.ED.A97K.01A | 0 | 6 | HIGH | 20073 | male | stage iii | G2 | T3 | N0 | M0 | 1.79476468 |
| TCGA.DD.AAD6.01A | 0 | 672 | HIGH | 24185 | male | stage iii | G3 | T3 | N0 | M0 | 1.87529641 |
| TCGA.ED.A8O6.01A | 1 | 56 | HIGH | 18572 | female | stage iii | G3 | T3 | N0 | M0 | 2.8448391 |
| TCGA.G3.AAV6.01A | 1 | 65 | HIGH | 19556 | female | stage iii | G3 | T3 | N0 | M0 | 3.23221105 |
| TCGA.G3.A6UC.01A | 0 | 671 | LOW | 23793 | male | stage iii | G2 | T3 | N0 | M0 | 1.07303793 |
| TCGA.DD.A4NH.01A | 0 | 917 | HIGH | 23865 | female | stage iii | G3 | T3 | N0 | M0 | 1.96803081 |
| TCGA.DD.A3A7.01A | 1 | 419 | HIGH | 24599 | male | stage iii | G3 | T3 | N0 | M0 | 2.57180433 |
| TCGA.PD.A5DF.01A | 1 | 639 | HIGH | 21414 | female | stage iii | G2 | T4 | N0 | M0 | 2.0712017 |
| TCGA.CC.A5UE.01A | 1 | 272 | HIGH | 17805 | male | stage iii | G2 | T4 | N0 | M0 | 3.30933562 |
| TCGA.CC.5260.01A | 1 | 87 | LOW | 22345 | female | stage iii | G1 | T4 | N0 | M0 | 1.35705718 |
| TCGA.MI.A75E.01A | 0 | 507 | HIGH | 22457 | male | stage iii | G2 | T4 | N0 | M0 | 1.65409085 |
| TCGA.CC.5262.01A | 1 | 103 | HIGH | 24787 | male | stage iii | G1 | T4 | N0 | M0 | 1.72021271 |
| TCGA.CC.5259.01A | 0 | 250 | HIGH | 21946 | female | stage iii | G2 | T4 | N0 | M0 | 1.73358859 |
| TCGA.BC.A8YO.01A | 0 | 562 | HIGH | 24189 | female | stage iii | G3 | T4 | N0 | M0 | 2.07895549 |
| TCGA.G3.AAV1.01A | 1 | 359 | HIGH | 18973 | male | stage iii | G3 | T4 | N0 | M0 | 3.11100077 |
| TCGA.DD.A1EJ.01A | 1 | 1005 | HIGH | 26009 | female | stage iii | G2 | T1 | N1 | M0 | 2.70645015 |
| TCGA.RC.A6M5.01A | 0 | 15 | HIGH | 7634 | female | stage iv | G2 | T1 | N1 | M0 | 1.81596184 |
| TCGA.DD.A4NA.01A | 0 | 1008 | HIGH | 24603 | female | stage iii | G3 | T2 | N1 | M0 | 1.84381283 |
| TCGA.CC.A8HS.01A | 1 | 300 | LOW | 6617 | male | stage iii | G1 | T3 | N1 | M0 | 1.58961712 |
| TCGA.DD.A114.01A | 1 | 1149 | LOW | 15410 | male | stage ii | G3 | T2 | NA | M0 | 1.11835473 |
| TCGA.MI.A75H.01A | 0 | 747 | LOW | 28371 | male | NA | NA | NA | NA | NA | 1.39421067 |
| TCGA.UB.AA0V.01A | 0 | 314 | LOW | 25340 | female | stage i | G1 | NA | NA | NA | 0.51805994 |
| TCGA.ZP.A9D0.01A | 0 | 1091 | LOW | 24525 | female | NA | G1 | T1 | NA | NA | 0.69931701 |
| TCGA.ZP.A9CV.01A | 1 | 1088 | LOW | 21633 | male | NA | G1 | T1 | NA | NA | 0.88479948 |
| TCGA.ZP.A9CY.01A | 0 | 782 | LOW | 24223 | female | NA | G1 | T1 | NA | NA | 0.99457011 |
| TCGA.ZP.A9D4.01A | 0 | 395 | LOW | 23656 | female | NA | G1 | T1 | NA | NA | 1.305061 |
| TCGA.ZP.A9CZ.01A | 0 | 706 | LOW | 26403 | male | NA | G1 | T1 | NA | NA | 1.38038925 |
| TCGA.ZP.A9D1.01A | 0 | 21 | HIGH | 20584 | female | NA | G2 | T1 | NA | NA | 1.68249636 |
| TCGA.2Y.A9H8.01A | 1 | 633 | HIGH | 31322 | female | NA | G2 | T1 | NA | NA | 2.05474339 |
| TCGA.2Y.A9GT.01A | 1 | 1624 | LOW | 18768 | male | stage i | G2 | T1 | NA | NA | 0.45302464 |
| TCGA.2Y.A9H6.01A | 0 | 357 | LOW | 24982 | female | stage i | G2 | T1 | NA | NA | 0.49034073 |
| TCGA.G3.A7M8.01A | 0 | 430 | LOW | 11527 | male | stage i | G1 | T1 | NA | NA | 0.55633044 |
| TCGA.K7.A5RF.01A | 0 | 631 | LOW | 23557 | male | stage i | G1 | T1 | NA | NA | 0.58675942 |
| TCGA.2Y.A9GV.01A | 1 | 2532 | LOW | 20011 | female | stage i | G1 | T1 | NA | NA | 0.75556453 |
| TCGA.MR.A520.01A | 0 | 229 | LOW | 21398 | male | stage i | G1 | T1 | NA | NA | 0.82693396 |
| TCGA.XR.A8TC.01A | 0 | 1339 | LOW | 15817 | female | stage i | G2 | T1 | NA | NA | 0.83332362 |
| TCGA.NI.A8LF.01A | 0 | 799 | LOW | 27162 | male | stage i | G3 | T1 | NA | NA | 0.84172352 |
| TCGA.O8.A75V.01A | 0 | 538 | LOW | 19850 | male | stage i | G2 | T1 | NA | NA | 0.87862713 |
| TCGA.2Y.A9GU.01A | 0 | 1939 | LOW | 20187 | female | stage i | G2 | T1 | NA | NA | 0.88583286 |
| TCGA.2Y.A9GX.01A | 0 | 2442 | LOW | 24963 | male | stage i | G2 | T1 | NA | NA | 1.03941609 |
| TCGA.EP.A12J.01A | 0 | 570 | LOW | 22997 | male | stage i | G1 | T1 | NA | NA | 1.09646791 |
| TCGA.WX.AA44.01A | 0 | 615 | LOW | 23654 | female | stage i | G3 | T1 | NA | NA | 1.16073906 |
| TCGA.GJ.A3OU.01A | 0 | 879 | LOW | 21671 | male | stage i | G2 | T1 | NA | NA | 1.17360694 |
| TCGA.UB.A7ME.01A | 0 | 486 | LOW | 18846 | male | stage i | G2 | T1 | NA | NA | 1.1800119 |
| TCGA.2Y.A9H1.01A | 1 | 1229 | LOW | 21222 | male | stage i | G2 | T1 | NA | NA | 1.31901417 |
| TCGA.K7.A5RG.01A | 0 | 519 | LOW | 24218 | male | stage i | G1 | T1 | NA | NA | 1.41723916 |
| TCGA.EP.A2KB.01A | 1 | 596 | LOW | 16835 | female | stage i | G2 | T1 | NA | NA | 1.41923672 |
| TCGA.2Y.A9HB.01A | 0 | 260 | LOW | 24359 | male | stage i | G2 | T1 | NA | NA | 1.43141731 |
| TCGA.EP.A3JL.01A | 0 | 303 | LOW | 27886 | male | stage i | G2 | T1 | NA | NA | 1.55249478 |
| TCGA.FV.A3R3.01A | 1 | 366 | LOW | 14231 | female | stage i | G2 | T1 | NA | NA | 1.5708307 |
| TCGA.ES.A2HS.01A | 1 | 688 | HIGH | 29268 | male | stage i | G2 | T1 | NA | NA | 1.65398971 |
| TCGA.FV.A3R2.01A | 1 | 194 | HIGH | 27530 | male | stage i | NA | T1 | NA | NA | 1.66562676 |
| TCGA.ES.A2HT.01A | 1 | 438 | HIGH | 20023 | male | stage i | G2 | T1 | NA | NA | 1.67035383 |
| TCGA.G3.A7M7.01A | 0 | 361 | HIGH | 24002 | male | stage i | G1 | T1 | NA | NA | 1.67286762 |
| TCGA.G3.A7M5.01A | 0 | 447 | HIGH | 27963 | male | stage i | G2 | T1 | NA | NA | 1.87511048 |
| TCGA.DD.AADN.01A | 0 | 898 | HIGH | 21632 | male | stage i | G4 | T1 | NA | NA | 2.04964006 |
| TCGA.EP.A2KC.01A | 1 | 19 | HIGH | 22677 | male | stage i | G3 | T1 | NA | NA | 2.07636402 |
| TCGA.WJ.A86L.01A | 0 | 345 | HIGH | 25018 | female | stage i | G2 | T1 | NA | NA | 2.15558858 |
| TCGA.XR.A8TF.01A | 1 | 693 | HIGH | 27145 | male | stage i | G1 | T1 | NA | NA | 2.80933419 |
| TCGA.G3.A7M6.01A | 0 | 632 | HIGH | 22205 | female | stage i | G3 | T1 | NA | NA | 2.96681436 |
| TCGA.BD.A2L6.01A | 0 | 1363 | LOW | 25489 | male | NA | G2 | T2 | NA | NA | 0.60015106 |
| TCGA.BC.A10Q.01A | 1 | 1135 | HIGH | 26400 | female | NA | NA | T2 | NA | NA | 1.71372838 |
| TCGA.MI.A75I.01A | 0 | 630 | HIGH | 22341 | male | NA | G1 | T2 | NA | NA | 2.14981237 |
| TCGA.ZP.A9D2.01A | 1 | 765 | HIGH | 18717 | male | NA | G2 | T2 | NA | NA | 2.5680213 |
| TCGA.2Y.A9GS.01A | 1 | 724 | HIGH | 21318 | male | NA | G2 | T2 | NA | NA | 2.60764134 |
| TCGA.BC.A10U.01A | 1 | 837 | HIGH | 25305 | male | NA | G2 | T2 | NA | NA | 2.63058268 |
| TCGA.BD.A3ER.01A | 0 | 1115 | LOW | 22657 | male | stage ii | G2 | T2 | NA | NA | 0.59675308 |
| TCGA.UB.AA0U.01A | 0 | 327 | LOW | 21985 | male | stage ii | G2 | T2 | NA | NA | 0.77862597 |
| TCGA.WX.AA46.01A | 0 | 756 | LOW | 22639 | male | stage ii | G1 | T2 | NA | NA | 0.83591197 |
| TCGA.2Y.A9H3.01A | 0 | 1516 | LOW | 16443 | male | stage ii | G1 | T2 | NA | NA | 1.17464687 |
| TCGA.ZS.A9CE.01A | 0 | 1241 | LOW | 29051 | female | stage ii | G1 | T2 | NA | NA | 1.48087373 |
| TCGA.ZS.A9CF.01A | 0 | 2412 | LOW | 23510 | male | stage ii | G2 | T2 | NA | NA | 1.56194027 |
| TCGA.ZS.A9CG.01A | 0 | 341 | HIGH | 20100 | male | stage ii | G2 | T2 | NA | NA | 1.71722054 |
| TCGA.2Y.A9GZ.01A | 1 | 848 | HIGH | 30082 | female | stage ii | G2 | T2 | NA | NA | 2.01461993 |
| TCGA.DD.A73D.01A | 0 | 693 | HIGH | 24941 | female | stage ii | G1 | T2 | NA | NA | 2.15969776 |
| TCGA.2Y.A9GY.01A | 1 | 757 | HIGH | 23435 | female | stage ii | G3 | T2 | NA | NA | 2.18931567 |
| TCGA.UB.A7MB.01A | 0 | 601 | HIGH | 8951 | male | stage ii | G3 | T2 | NA | NA | 2.19528838 |
| TCGA.ZS.A9CD.01A | 1 | 1386 | HIGH | 26915 | male | stage ii | G2 | T2 | NA | NA | 2.28656954 |
| TCGA.QA.A7B7.01A | 0 | 94 | HIGH | 17636 | male | stage ii | G2 | T2 | NA | NA | 2.5264055 |
| TCGA.2Y.A9HA.01A | 1 | 36 | HIGH | 25892 | male | stage ii | G2 | T2 | NA | NA | 2.74991831 |
| TCGA.4R.AA8I.01A | 1 | 262 | HIGH | 24279 | male | stage ii | G2 | T2 | NA | NA | 2.91481115 |
| TCGA.GJ.A6C0.01A | 1 | 31 | HIGH | 27626 | female | stage ii | G2 | T2 | NA | NA | 3.38557093 |
| TCGA.K7.AAU7.01A | 0 | 359 | HIGH | 22476 | male | stage ii | G2 | T2 | NA | NA | 1.90961703 |
| TCGA.BC.A10R.01A | 1 | 308 | HIGH | 24274 | female | NA | G2 | T3 | NA | NA | 1.81810068 |
| TCGA.NI.A4U2.01A | 1 | 1791 | HIGH | 26011 | male | stage iii | G1 | T3 | NA | NA | 1.91747336 |
| TCGA.RC.A6M4.01A | 0 | 22 | HIGH | 27327 | female | stage iii | G2 | T3 | NA | NA | 2.03445163 |
| TCGA.UB.A7MF.01A | 1 | 214 | HIGH | 20815 | male | stage iii | G2 | T3 | NA | NA | 1.79262088 |
| TCGA.EP.A3RK.01A | 0 | 363 | HIGH | 26985 | male | stage iii | G2 | T3 | NA | NA | 2.07192657 |
| TCGA.BW.A5NO.01A | 0 | 20 | HIGH | 18510 | male | stage iii | G2 | T3 | NA | NA | 2.13145741 |
| TCGA.WX.AA47.01A | 1 | 556 | HIGH | 12056 | female | stage iii | G2 | T3 | NA | NA | 2.3191719 |
| TCGA.EP.A2KA.01A | 1 | 627 | HIGH | 19214 | female | stage iii | G3 | T3 | NA | NA | 2.94019084 |
| TCGA.3K.AAZ8.01A | 0 | 396 | LOW | 24046 | male | stage iii | G1 | T3 | NA | NA | 1.09923861 |
| TCGA.G3.A7M9.01A | 1 | 56 | HIGH | 25588 | male | stage iii | G2 | T3 | NA | NA | 3.47589902 |
| TCGA.BC.A10Y.01A | 1 | 711 | HIGH | 28049 | male | NA | G3 | T4 | NA | NA | 1.84549992 |
| TCGA.BC.A10T.01A | 1 | 837 | HIGH | 27944 | male | NA | G1 | T4 | NA | NA | 2.4152758 |
| TCGA.BC.A10W.01A | 1 | 91 | HIGH | 18562 | male | NA | G3 | T4 | NA | NA | 3.7160676 |
| TCGA.T1.A6J8.01A | 0 | 23 | LOW | 24853 | male | NA | G2 | T1 | NA | M0 | 1.11102285 |
| TCGA.BC.A3KF.01A | 0 | 8 | LOW | 24328 | female | stage i | G2 | T1 | NA | M0 | 0.75682307 |
| TCGA.FV.A4ZQ.01A | 0 | 12 | LOW | 19090 | male | stage i | G2 | T1 | NA | M0 | 0.80342881 |
| TCGA.G3.A5SK.01A | 0 | 744 | LOW | 21320 | male | stage i | G1 | T1 | NA | M0 | 0.92261136 |
| TCGA.ED.A627.01A | 0 | 423 | LOW | 27303 | male | stage i | G2 | T1 | NA | M0 | 1.24339602 |
| TCGA.HP.A5MZ.01A | 1 | 91 | LOW | 22702 | male | stage i | G2 | T1 | NA | M0 | 1.54154283 |
| TCGA.XR.A8TG.01A | 0 | 898 | HIGH | 21544 | male | stage i | G2 | T1 | NA | M0 | 1.74359611 |
| TCGA.LG.A9QC.01A | 0 | 425 | HIGH | 17842 | male | stage i | G2 | T1 | NA | M0 | 1.7539549 |
| TCGA.G3.A5SJ.01A | 0 | 698 | HIGH | 21848 | male | stage i | G2 | T1 | NA | M0 | 1.78464008 |
| TCGA.FV.A496.01A | 0 | 10 | HIGH | 30950 | female | stage i | G2 | T1 | NA | M0 | 2.27123148 |
| TCGA.DD.A39X.01A | 1 | 1694 | HIGH | 28528 | female | stage i | G2 | T1 | NA | M0 | 2.44680875 |
| TCGA.DD.A4NI.01A | 0 | 816 | LOW | 24516 | male | stage ii | G2 | T2 | NA | M0 | -0.1065052 |
| TCGA.ED.A7PY.01A | 0 | 390 | LOW | 7556 | female | stage ii | G3 | T2 | NA | M0 | 0.17612028 |
| TCGA.DD.A39Z.01A | 1 | 601 | LOW | 15930 | female | stage ii | G2 | T2 | NA | M0 | 1.24856592 |
| TCGA.FV.A495.01A | 0 | 1 | LOW | 18740 | female | stage ii | G2 | T2 | NA | M0 | 1.53386766 |
| TCGA.FV.A3I0.01A | 0 | 848 | LOW | 28101 | female | stage ii | G2 | T2 | NA | M0 | 1.55273022 |
| TCGA.G3.A5SL.01A | 0 | 621 | HIGH | 25671 | male | stage ii | G2 | T2 | NA | M0 | 1.59059199 |
| TCGA.ED.A7PX.01A | 0 | 6 | HIGH | 17638 | female | stage ii | G3 | T2 | NA | M0 | 1.61343405 |
| TCGA.WQ.AB4B.01A | 0 | 395 | HIGH | 22769 | male | stage ii | G2 | T2 | NA | M0 | 1.69157339 |
| TCGA.G3.A5SM.01A | 0 | 520 | HIGH | 21236 | male | stage ii | G3 | T2 | NA | M0 | 1.80907044 |
| TCGA.BC.A69H.01A | 0 | 444 | HIGH | 23380 | male | stage ii | G3 | T2 | NA | M0 | 2.10812381 |
| TCGA.LG.A6GG.01A | 0 | 387 | HIGH | 28871 | female | stage ii | G2 | T2 | NA | M0 | 2.46082595 |
| TCGA.DD.A39V.01A | 1 | 643 | HIGH | 28254 | male | stage ii | G3 | T2 | NA | M0 | 2.61558992 |
| TCGA.BC.A217.01A | 1 | 1397 | HIGH | 27411 | female | stage ii | G3 | T2 | NA | M0 | 2.72886274 |
| TCGA.RC.A6M6.01A | 0 | 9 | HIGH | 27649 | male | stage ii | G3 | T2 | NA | M0 | 3.23005688 |
| TCGA.ED.A7PZ.01A | 0 | 6 | HIGH | 22601 | male | stage ii | G2 | T2 | NA | M0 | 3.41691585 |
| TCGA.FV.A4ZP.01A | 1 | 2486 | HIGH | 28771 | male | stage iii | G2 | T3 | NA | M0 | 1.93271605 |
| TCGA.BC.A216.01A | 0 | 1351 | HIGH | 22968 | female | stage iii | G2 | T3 | NA | M0 | 2.61406788 |
| TCGA.WQ.A9G7.01A | 0 | 30 | LOW | NA | female | NA | G3 | T3 | NA | M0 | 1.24673236 |
| TCGA.BC.A5W4.01A | 1 | 547 | LOW | 25503 | male | stage iii | G3 | T3 | NA | M0 | 1.42286649 |
| TCGA.DD.A4NG.01A | 1 | 802 | HIGH | 28234 | male | stage iii | G2 | T3 | NA | M0 | 1.92082168 |
| TCGA.HP.A5N0.01A | 1 | 752 | LOW | 32120 | female | NA | NA | NA | NA | M0 | 1.07377047 |
